# Synthesis and reactivity of low-valent, high-spin 2 : 1 bisphosphine–iron complexes

**DOI:** 10.1039/d6cc06766b

**Published:** 2026-11-03

**Authors:** Egor Kokin, Shih-Chieh Kao, Michael L. Neidig

**Affiliations:** a Inorganic Chemistry Laboratory, Department of Chemistry, University of Oxford Oxford UK michael.neidig@chem.ox.ac.uk

## Abstract

Herein we report the synthesis and characterisation of the first high-spin 2 : 1 bisphosphine–Fe(i) (*S* = 3/2) and Fe(0) (*S* = 1) complexes supported by 1,2-bis(dicyclohexylphosphino)ethane (dcype). Their fate under catalytic conditions is explored and the reactivity studies reveal limited reactivity of the Fe(i) complex towards an electrophile in the absence of a nucleophile.

## Abstract 2

In this work, we report the synthesis, characterisation, and reactivity of the first examples of high-spin 2 : 1 Fe(i) and Fe(0) bisphosphine complexes.

## Graphical abstract

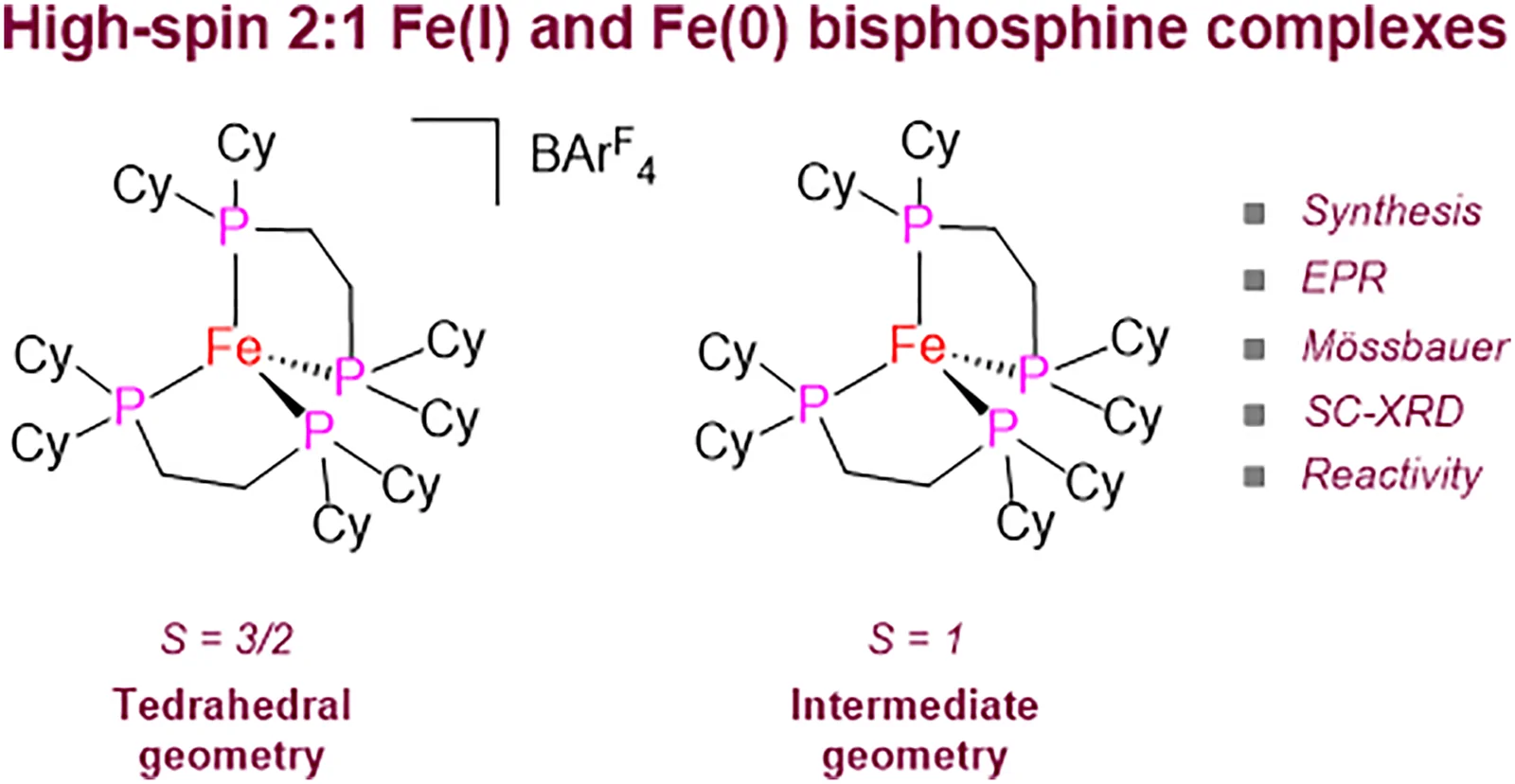

Low-valent iron complexes are proposed to play central roles in multiple catalytic transformations, ranging from classical cross-coupling^1–3^ and C–H activation reactions^4–6^ to nitrogen activation.^7–9^ Bisphosphine ligands occupy a privileged role in many of these reactions, enabling high-reactivity and good catalytic performance. For the latter, extra bisphosphine ligand is often added to the catalytic mixture to improve the overall yields.^2,10–12^ The importance of an excess ligand remains a debated topic in the field. On the one hand, the ligand can stabilise the low-valent species that are essential for catalysis, as suggested in the Fe^II/III/I^ cycle widely accepted as the most common mechanism in the cross-coupling reactions with iron–bisphosphines.^1–3^ On the other hand, the extra equivalent of the ligand can simply prevent the formation of the over-transmetalated Fe(ii) species that are detrimental to catalysis.^13^

In the area of cross-coupling, several key studies have sought to address the role of potential 2 : 1 bisphosphine–Fe complexes that could form in the presence of excess bisphosphine ligand, particularly low-valent complexes. For example, while Bedford and co-workers demonstrated that low-valent 2 : 1 complexes with 1,2-bis(diphenylphosphino)ethane (dppe) and 1,2-bis(diphenylphosphino)benzene (dppbz) can be formed and serve as efficient precatalysts for cross-coupling reactions (Scheme 1),^14–16^ kinetic studies suggested that the on-cycle species form from these complexes *via* ligand dissociation.^17^ More recently, Townrow has reported several 2 : 1 low-spin complexes with bisphosphine ligands and demonstrated their catalytic competence as precatalysts in an aryl–alkyl Kumada cross-coupling reaction.^18^ However, similar aryl–alkyl Kumada cross-couplings can readily be catalysed by a simple mixture of an Fe(ii) salt and bisphosphine ligand, and the actual fate of the reduced 2 : 1 bisphosphine–Fe complex upon entering the catalytic cycle remains unknown.^12^

**Scheme 1:**
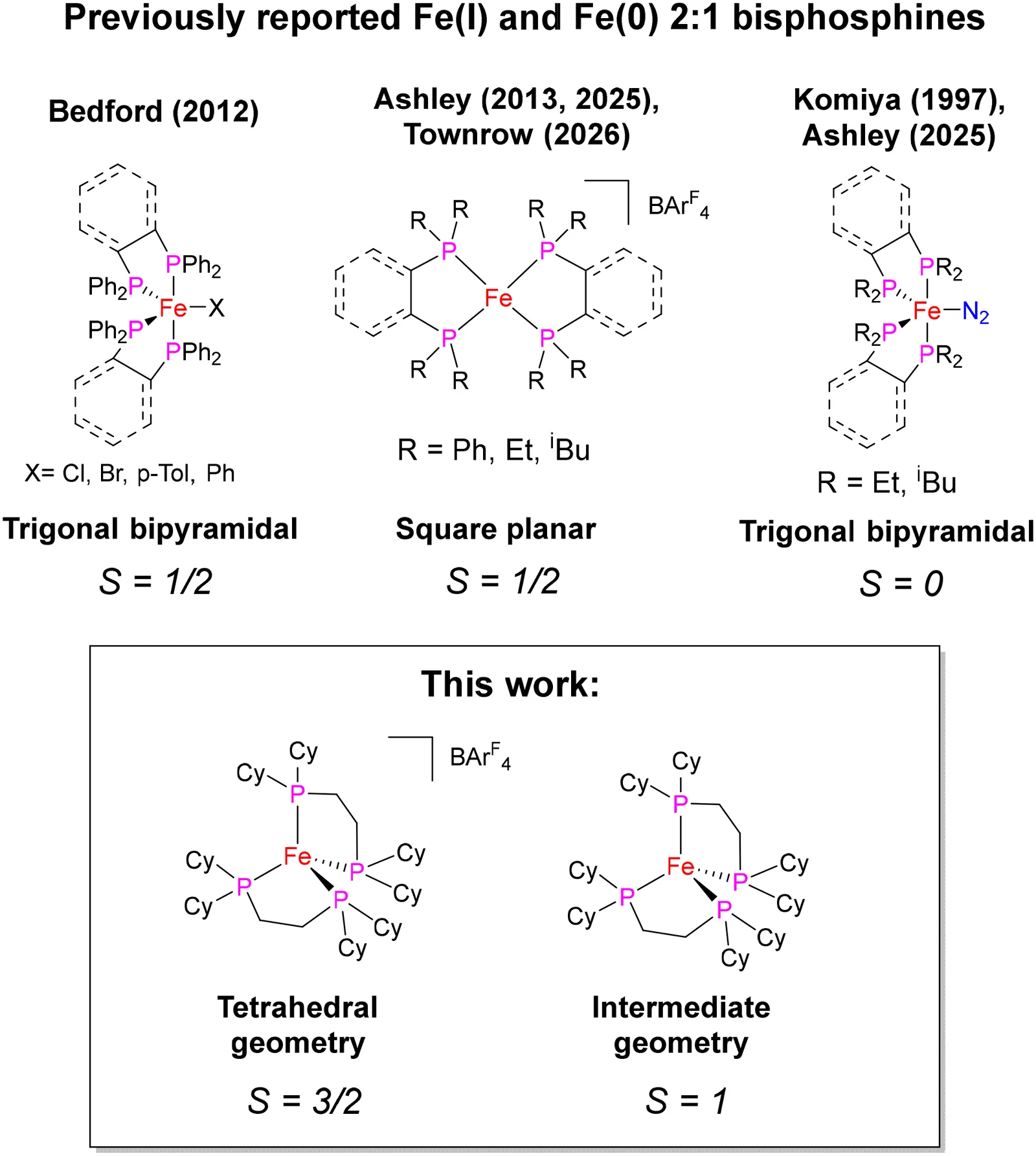
Representative examples of the low-valent 2 : 1 bisphosphine–iron complexes and the novel complexes introduced in this work.

Furthermore, despite these prior studies it is notable that the 2 : 1 bisphosphine–Fe(i) complexes reported to date all have *S* = 1/2 ground states in either trigonal bipyramidal (if additional ligand X = Cl, Br, alkyl, or aryl is present)^14–16^ or square planar geometries (if the charge is balanced by a non-coordinating anion) (Scheme 1).^8,19^ The high-spin (*S* = 3/2) state remains unknown for these types of complexes.

Beyond cross-coupling, the isolation of 2 : 1 bisphosphine–Fe(0) complexes, highly reactive complexes for CH-activation, ^20^ is of substantial interest yet there are no examples of structurally and spectroscopically characterised four-coordinate 2 : 1 bisphosphine : Fe(0) complexes. Only CH-activated iron products or higher-coordinate dinitrogen adducts can be structurally characterised,^9^ and, to the best of our knowledge, akin to the corresponding Fe(i) complexes previously discussed the high-spin adducts remain unknown for this class of complexes.

To address these limitations in the field, we focused on the synthesis of the 2 : 1 dcype complex *via* KC_8_ reduction, which has proven to be a successful approach for accessing low-oxidation state iron complexes.^21^ The dcype ligand was selected both due to its importance in iron-catalysed cross-coupling reactions, as well as its identified preference for lower coordination number complexes.^2,21^ NaBAr^F^_4_ was included in the reaction as a source of a non-coordinating anion. Upon addition of 2.5 equiv. of KC_8_ to a mixture of (dcype)FeBr_2_ (1), dcype ligand (1 equiv.) and NaBAr^F^_4_ (1.1 equiv.) at −78 °C in 2-MeTHF, a colour change of the solution from colourless to deep green was observed (Fig. S3). After stirring the reaction mixture at −78 °C for an additional 10 min, the deep blue colour indicative of the square-planar Fe(i) 2 : 1 complex develops (Fig. S4),^8,18,19^ Recrystallisation from 2-MeTHF/pentane mixture at −35 °C, yielded colourless crystals of the product in moderate yield (53%). SC-XRD^22^ revealed the structure as [(dcype)_2_Fe][BAr^F^_4_] (2) with the Fe centre in a distorted tetrahedral environment (*τ*_4_ = 0.82, Fig. 1A), contrary to the previously reported 2 : 1 bisphosphate–Fe(i) complexes with 1,2-bis(diethylphosphino)ethane (depe), 1,2-bis(diisobutylphosphino)ethane (dibpe), dppe, and dppbz, which are shown to be square planar.^8,18,19^ The compound crystallises in the orthorhombic space group *Pnna*, with both Fe and B in special positions. Two halves of the anion and cation are generated by the two-fold axes, therefore maintaining the perfectly staggered arrangement of the phosphine ligands. Fe–P bonds are notably long (2.3854(7)–2.3695(7) Å, average 2.377 Å) compared to the previously reported [(dcype)Fe(µ-Br)]_2_ (average Fe–P = 2.294 Å) and square planar [(dppe)_2_Fe][BAr^F^_4_] (average Fe–P = 2.227 Å) possibly due to the steric repulsion between bulky cyclohexyl groups.

**Fig. 1:**
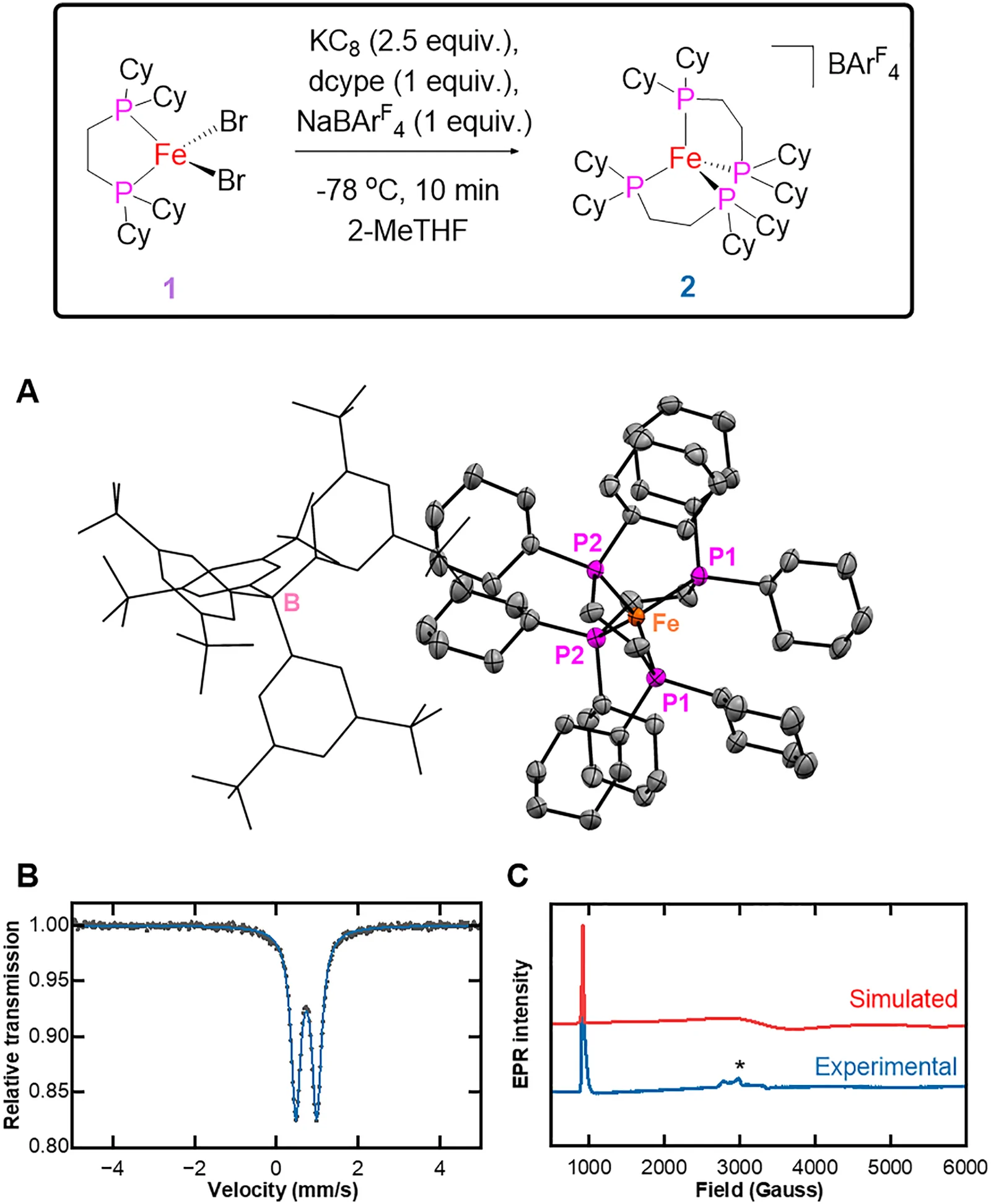
Synthesis and characterisation of [(dcype)_2_Fe][BAr^F^_4_] (2). (A) X-ray crystal structure of 2 with thermal ellipsoids drawn at the 50% probability level. Hydrogen atoms and co-crystallised solvent have been removed for clarity, and the BAr^F^_4_^−^ counterion is shown in wireframe. (B) 80 K ^57^Fe Mössbauer spectrum of solid 2, characterised by a doublet with *δ* = 0.78 mm s^−1^ and |Δ*E*_Q_| = 0.53 mm s^−1^. (C) 10 K EPR spectrum of the 2-MeTHF frozen solution of 2. The spectrum was simulated with an *S* = 3/2 Hamiltonian with parameters: *g*_1,real_ = 2.10, *g*_2,real_ = 2.00 *g*_3,real_ = 2.45, *λ* = *E*/*D* = 0.12, *D* = −12 cm^−1^. Note: the asterisk marks an *S* = 1/2 signal corresponding to the square planar complex analogous to the previously reported.^19^

Intrigued by the complex's unusual geometry, we decided to pursue a full spectroscopic characterisation to gain insight into its electronic structure and bonding. Solid and frozen-solution ^57^Fe Mössbauer spectra of 2 are characterised by a quadrupolar doublet with parameters: *δ* = 0.78 mm s^−1^ and |Δ*E*_Q_| = 0.53 mm s^−1^, and *δ* = 0.78 mm s^−1^ and |Δ*E*_Q_| = 0.53 mm s^−1^, respectively (Fig. 1B and Fig. S3). The identical parameters indicate that the tetrahedral geometry is preserved in solution. 10 K EPR reveals the appearance of an *S* = 3/2 signal that can be described as a fictitious *S* = 1/2 with a highly anisotropic *g* tensor: *g*_1,effective_ = 7.23, *g*_2,effective_ = 1.01, and *g*_3,effective_ = 1.01 (Fig. S15). These values are typical for |*m*_s_ = ±3/2〉 Kramers doublet of *S* = 3/2 spin systems with large (|*D*| ≫ 0.3 cm^−1^), rhombic zero field splitting and *D* < 0.^23,24^ The signal can also be adequately fit with a *S* = 3/2 Hamiltonian with parameters: *g*_1,real_ = 2.10, *g*_2,real_ = 2.00, *g*_3,real_ = 2.45, *λ* = *E*/*D* = 0.12, *D* = −12 cm^−1^ (Fig. 1C). Interestingly, an additional very minor *S* = 1/2 feature is observed, which contributes less than 1% to the total intensity and was simulated with parameters: *g*_1_ = 2.42, *g*_2_ = 2.23, *g*_3_ = 1.99, and hyperfine coupling to four equivalent ^31^P sites with constants *A*_2_ = 55.4 and *A*_3_ = 55.5 MHz (Fig. S16). This minor *S* = 1/2 signal is attributed to the square-planar complex, given the similarity of the EPR parameters to those previously reported for [(depe)_2_Fe][BAr^F^_4_].^19^ Even though *S* = 1/2 species represents less than 1% of the total intensity, the presence of this signal indicates that the structural transformation between tetrahedral and square-planar conformations is possible in solution. By contrast, the *S* = 1/2 signal does not appear in the solid state, with only the *S* = 3/2 signal observed (Fig. S17). The magnetic moment measurements of 2 in the solid state (*µ*_eff_ = 4.3(2) µB, Fig. S22)), further corroborates the *S* = 3/2 ground state of the complex.

Next, we investigated the possibility of further reduction of 2. Cyclic voltammetry (CV) revealed the presence of the reversible redox process with *E* = −2.34 V *vs.* Fc^+^/Fc (Fig. S1 and S2). This observation is attributed to the Fe(i)/Fe(0) couple and indicates that a Fe(0) complex should be accessible. Mg metal has previously been used in synthesis of [(dcype)Fe(µ-Br)]_2_ complex with the similar redox potential.^21^ Therefore, we decided to use this mild reductant in our synthesis. Overnight reaction of (dcype)FeBr_2_ with an excess of Mg powder and an extra equivalent of dcype led to a colour change to dark orange (Fig. S6). And the orange crystalline product was isolated from this reaction and identified as (dcype)_2_Fe 3. Alternatively, the same product can be accessed by reduction of 2 with 1.2 equiv. of KC_8_. SC-XRD (Fig. 2A) reveals the presence of an Fe centre in an intermediate geometry (*τ*_4_ = 0.65) between tetrahedral and square planar in contrast to tetrahedral geometry of 2. Unfortunately, the Fe centre is disordered over two positions, therefore the bond metrics are not discussed in detail. Solid state ^57^Fe Mössbauer is characterised by a doublet with *δ* = 0.42 mm s^−1^ and |Δ*E*_Q_| = 1.12 mm s^−1^ consistent with the presence of a *S* = 1 Fe centre (Fig. 2B). The spin state is further corroborated by the solid state magnetometry (*µ*_eff_ = 3.0(2) *µ*_B_, spin only value *µ*_eff_ = 2.83 *µ*_B_) (Fig. S23). Due to the poor solubility at low temperatures and apparent instability at RT (a new iron species forms in solution with parameters *δ* = 0.79 mm s^−1^ and |Δ*E*_Q_| = 1.84 mm s^−1^ potentially due to C–H activation and iron(ii) product formation known for such complexes,^9,20^ Fig. S4) no further solution state characterisation was pursued. However, the isolated complex represents the first structurally characterised four-coordinate 2 : 1 bisphosphine–Fe(0) complex.

**Fig. 2:**
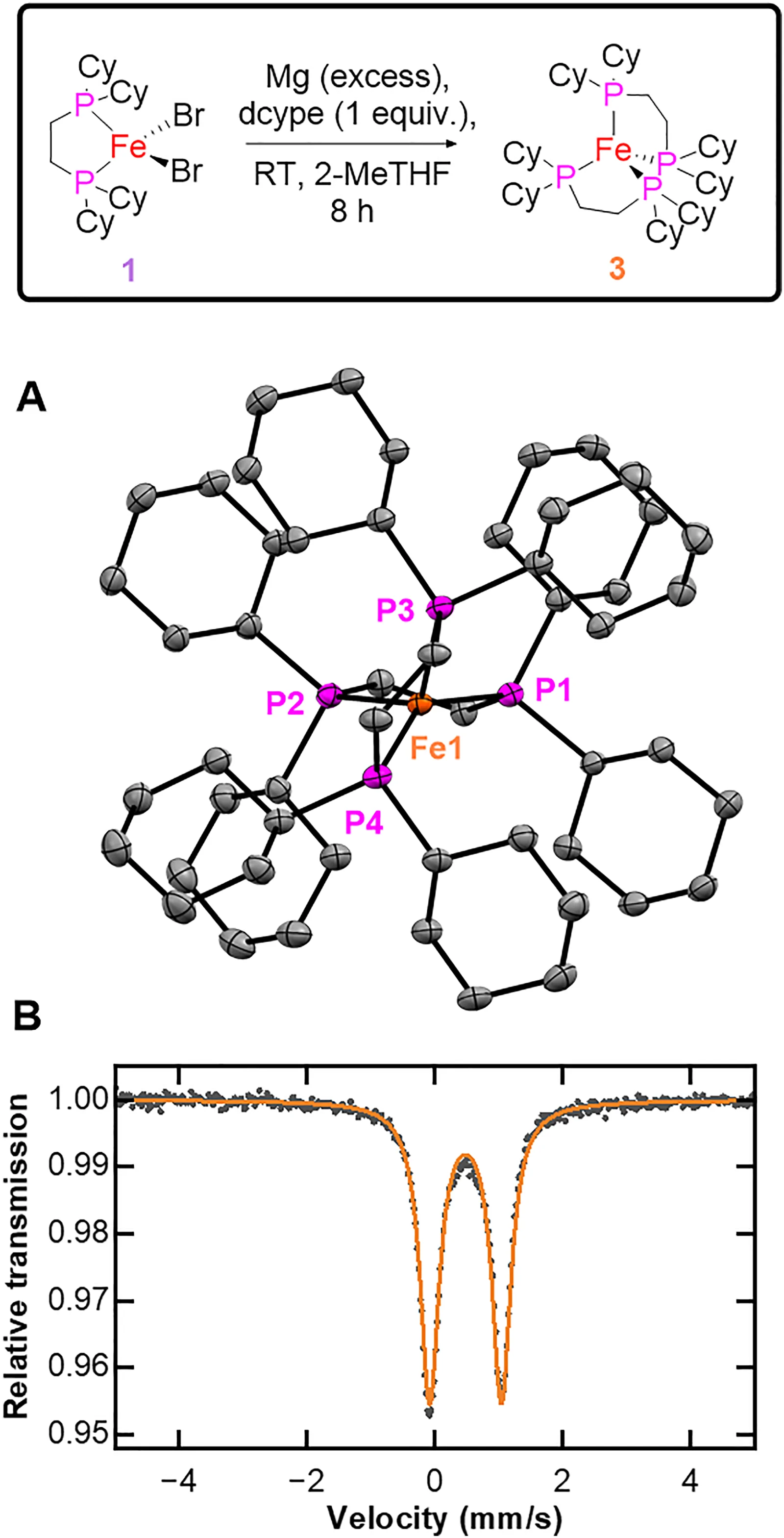
Synthesis and characterisation of (dcype)_2_Fe 3. (A) X-ray crystal structure of 3 with thermal ellipsoids drawn at the 50% probability level. Hydrogen atoms have been removed for clarity. (B) 80 K ^57^Fe Mössbauer spectrum of solid 3, characterised by a doublet with *δ* = 0.42 mm s^−1^ and |Δ*E*_Q_| = 1.12 mm s^−1^.

In terms of reactivity, 2 : 1 bisphosphine–Fe(i) complexes have proven effective as pre-catalysts in the Kumada cross-coupling reactions between alkyl halides and aryl Grignard reagents.^18^ The 2 : 1 complexes reported by Bedford and co-workers are reactive towards electrophiles and regenerate Fe(ii) complexes under catalytic conditions.^14–16^ However, similar reactivity has not been established for 4-coordinate 2 : 1 bisphosphine–Fe(i) complexes. Therefore, we decided to test the reactivity of 2 under catalytic conditions. 2 can serve as an efficient precatalyst for the cross-coupling of PhMgBr and CyBr, affording product in high yields (94% detected by NMR, Scheme S1). Despite this, complex 2 demonstrates limited reactivity towards electrophile, achieving 21% conversion within 1 hour (*k*_obs_ ≈ 0.033 min^−1^, 0 °C) with the formation of 20% of cyclohexane as reduced product (Scheme 2, Fig. S10) suggesting 2 is not an on-cycle reactive species. The presence of the excess ligand has no effect on the observed reaction rates (Fig. S11).

**Scheme 2:**
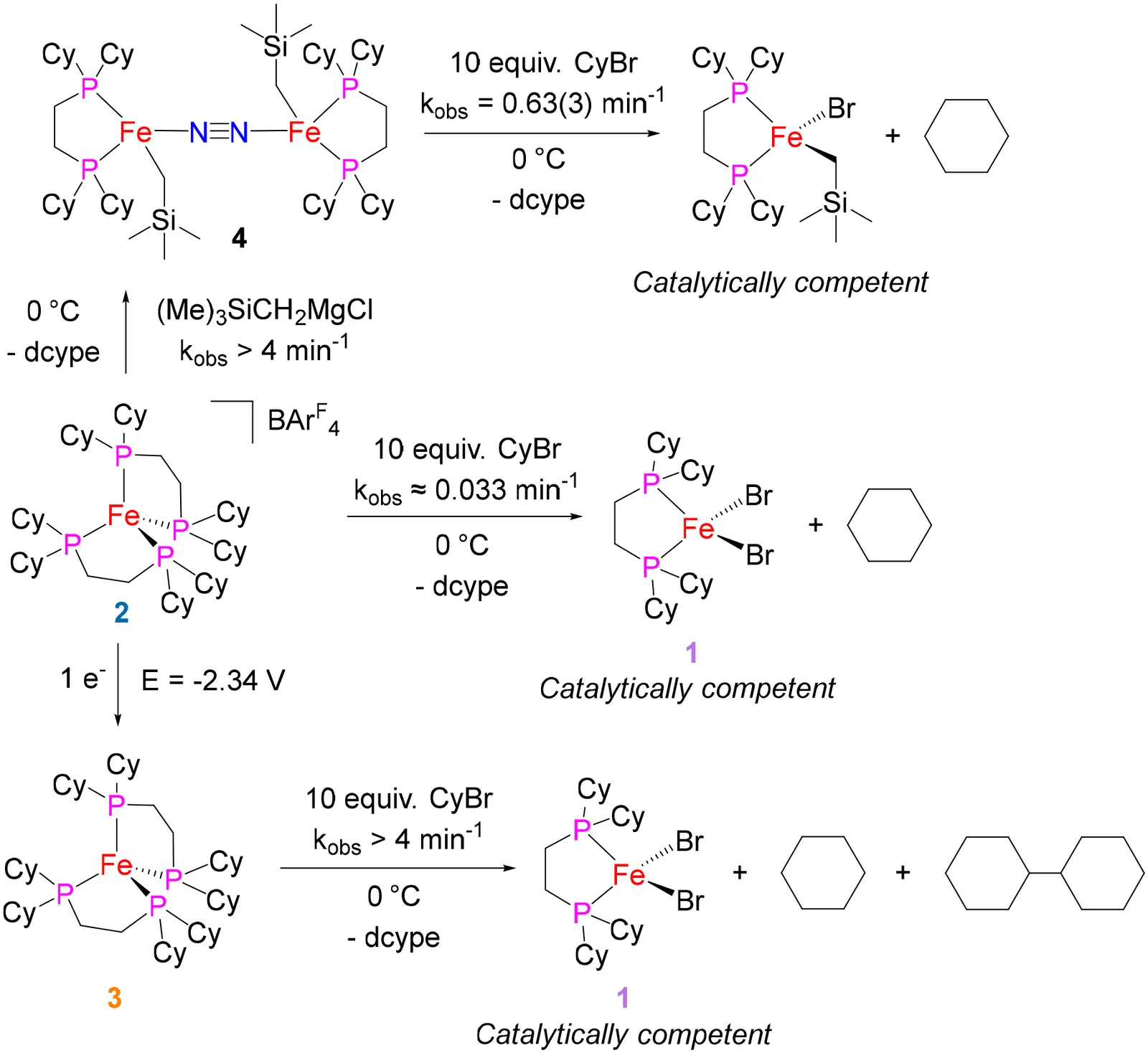
Reactivity of the low-valent 2 : 1 bisphosphine–Fe complexes 2 and 3. Note: Fe(ii) products are catalytically competent.^21^

On the other hand, 2 is highly reactive towards nucleophile (for example, (CH_3_)SiCH_2_MgCl), achieving complete conversion in less than 30 s (*k*_obs_ > 4 min^−1^, 0 °C) to the previously reported complex [(dcype)Fe(CH_2_Si(CH_3_)_3_]_2_[µ-N_2_] 4 (Scheme 2, Fig. S10) and the release of the free phosphine, as detected by ^31^P NMR (Fig. S20). Note that although (CH_3_)_3_SiCH_2_MgCl does not participate in iron-catalyzed cross-coupling reactions, we nevertheless chose it for our stoichiometric studies due to the stability of the transmetalated Fe(i) product.^21^2 does not react with PhMgBr at low temperature (−78 °C) (Fig. S8). However, at higher temperature (0 °C) the reaction occurs with the formation of the mixture of products, indicative of decomposition (Fig. S9). Once formed, the transmetalated product 4 is reactive towards electrophiles (*k*_obs_ = 0.63(3) min^−1^, 0 °C), regenerating Fe(ii), which can then continue the catalytic cycle as reported previously (Scheme 2).^21^

Unlike 2, 3 is much more reactive towards CyBr at 0 °C with the reaction complete in less than 30 s (*k*_obs_ > 4 min^−1^, 0 °C) (Scheme 2, Fig. S12). 1 was detected by freeze-trapped Mössbauer as the major iron product (81%). The formation of the free dcype was detected by ^31^P NMR (Fig. S16) which indicates that dissociation of the phosphine occurs during the reaction with the electrophile. The yields of the organic products (88% of cyclohexane and 30% of bicyclohexyl) are consistent with reaction of 3 with 2 equivalents of electrophile (Scheme 2). Similar to 2, excess ligand has no effect on the observed reactivity of 3 indicating that the dissociation of the ligand is not required for the reaction with electrophile.

To further confirm the importance of the nucleophile for the reactivity of low-valent 2 : 1 dcype–Fe(i) complexes with electrophile, we decided to perform a single turnover experiment with excess electrophile and 1 equivalent of PhMgBr added dropwise (Fig. S14). The quantitative formation of the cross-coupled product was observed by ^1^H NMR, and the freeze-trapped ^57^Fe Mössbauer reveal the formation of a single Fe(ii) product identified as 1. This confirms that the reactivity towards the electrophile is unlocked following either transmetallation or reduction by nucleophile and dissociation of the extra phosphine ligand.

In summary, we have synthesised the first example of a four-coordinate 2 : 1 bisphosphine–Fe complex with tetrahedral geometry and *S* = 3/2 ground state. The complex was fully characterised by ^57^Fe Mössbauer and EPR spectroscopies, as well as magnetic studies. The compound exhibited limited reactivity towards electrophiles in the absence of the Grignard reagent (Scheme 2). However, the catalytically competent species can be regenerated upon reaction with nucleophile either *via* transmetalation or reduction. The reduction reaction results in the formation of the novel tetracoordinate Fe(0) bisphosphine complex 3. The complex adopts distorted tetrahedral geometry in the solid state and *S* = 1 ground state. 3 rapidly reacts with catalytically relevant electrophile with the formation of the on-cycle complex 1 and reduced organic product. This work contributes to a better understanding of the structures and reactivity of low-valent iron–bisphosphine complexes that can form in catalysis and the possible reaction pathways that may allow seemingly unreactive overstabilised species to enter the catalytic cycle.

## Author contributions

Egor Kokin: investigation, conceptualisation, writing – original draft, writing – review and editing. Shih-Chieh Kao: writing – original draft, investigation, writing – review and editing. Michael L. Neidig: conceptualisation, funding acquisition, writing – review and editing, project administration, supervision.

## Conflicts of interest

The authors declare no conflicts of interest.

## Supplementary Material

CC-OLF-D6CC06766B-s001

## Data Availability

The data that support the findings of this study are available from the corresponding author upon reasonable request. Supplementary information (SI) includes full experimental details, cyclic voltametry (CV), ^57^Fe Mössbauer spectroscopy, continuous wave (CW) X-band EPR spectroscopy, NMR spectroscopy, magnetometry and crystallographic data. See DOI: https://doi.org/10.1039/d6cc06766b. CCDC 2588736 and 2588737 contain the supplementary crystallographic data for this paper.^22*a*,*b*^
