# Supplementary material for: Synthesis and reactivity of low-valent, high-spin 2 : 1 bisphosphine–iron complexes
Source: Chem Commun (Camb). 2026 Nov 3. Online ahead of print. doi: 10.1039/d6cc06766b (PMC13750519; doi:10.1039/d6cc06766b)
Supplement: Supplementary file 1 [file CC-OLF-D6CC06766B-s001.pdf]

Supplementary Information (SI) for Chemical Communications.
This journal is © The Royal Society of Chemistry

Supporting Information

Synthesis and Reactivity of Low-Valent, High-Spin 2:1 Bisphosphine-Iron Complexes

Egor Kokin, Shih-Chieh Kao and Michael L. Neidig*

Table of Contents

1. Experimental Procedures S3

1.1 General Considerations S3

1.2 Handling temperature- and air-sensitive crystals for X-ray diffraction S3

1.3 ^{57}Fe Mössbauer spectroscopy S3

1.4 Continuous wave (CW) X-band EPR spectroscopy S3

1.5 ^{1}H NMR and ^{31}P NMR Spectroscopy S3

1.6 Gas Chromatography S3

1.7 Solution-State Magnetic Susceptibility Measurement S3

1.8 Solid-State Magnetic Susceptibility Measurement S3

1.9 Synthetic procedures S4

2. Cyclic Voltammetry (CV) S6

3. Supplementary ^{57}Fe Mössbauer spectroscopy S7

4. Supplementary Continuous Wave (CW) X-band EPR spectroscopy S13

5. Supplementary NMR spectroscopy S15

6. Magnetometry Data S18

7. Supplementary Crystallographic Data S19

8. References S21

1. Experimental Procedures

1.1 General Considerations. All reagents not outlined herein were purchased from commercial sources. All air- and moisture-sensitive manipulations were carried out in an MBraun inert-atmosphere (N_2) glovebox equipped with a direct liquid nitrogen feed through an inlet line. All anhydrous solvents were freshly dried using activated alumina/4 Å molecular sieves and stored under an inert atmosphere. $^{57}FeBr_2$ was synthesised following literature procedures starting from ^{57}Fe metal (95% enriched) purchased from Isoflex. Unlabelled $FeBr_2$ was synthesised following the same procedure. [1] (dcype)$FeBr_2$ was synthesised as previously reported.

1.2 Handling temperature- and air-sensitive crystals for X-ray diffraction. Temperature-sensitive crystals were transferred to a nitrogen-purged glovebag in an aluminium pie block containing dry ice. A customised aluminium block containing inlet and outlet nozzles allowed for the passage of liquid nitrogen. The cold aluminium block was placed on the base of the microscope and was used to cool microscope slides containing SilOil. Aliquots of the cold crystalline material were placed on the cold microscope slides using liquid nitrogen-cooled pipettes. Single crystals were transferred from the microscope slide using a goniometer pin that was encased in liquid nitrogen-cooled aluminium.

1.3 ^{57}Fe Mössbauer spectroscopy. Solid and freeze-quenched solution samples were prepared in an MBraun inert-atmosphere N_2 glovebox equipped with a liquid nitrogen fill line, enabling sample freezing to 77 K within the glovebox. Mössbauer Spectra were collected with See Co. MS4 Mössbauer spectrometers integrated with a Janis SVT400T He/N_2 cryostat or using a WissEl spectrometer equipped with a DFG-1000 unit providing the Mössbauer Driving System with the reference signal for the triangular waveform of the source motion, MR-360 drive-unit to control the source motion, MVT-1000 velocity transducer, LND-45431 proportional counter, HVS-2 power supply, PEA-6 pre-amplifier, AMP-1000 main amplifier and CMCA-550 data acquisition module. The fitting of Mössbauer spectra was performed using WinNormos-for-Igor, with Isomer shifts determined relative to α-Fe at 298 K. Errors of the fit analysis were the following: $\delta \pm 0.02$ mm/s and $|\Delta E_Q| \pm 3\%$.

1.4 Continuous wave (CW) X-band EPR spectroscopy. Samples for EPR analysis were prepared in an inert atmosphere glovebox (N_2) equipped with a liquid nitrogen fill port to enable sample freezing to 77 K within the glovebox. EPR samples were prepared in 4 mm OD Suprasil quartz EPR tubes (Wilmad Labglass). X-band EPR spectra were recorded at 90 or 10 K on a Bruker EMX micro X-band continuous-wave EPR spectrometer with a super high sensitivity (SHQE) resonator and an ESR900 continuous-flow cryostat. EPR spectra were recorded at room temperature at a microwave frequency of ca. 9.74 GHz with modulation amplitudes of 10 G (for S = 3/2 species) or 1 G (for S = 1/2 species) and 100 kHz 100 kHz modulation frequency. The microwave power was scanned to rule out spectral distortions caused by saturation. The optimal microwave power used in all the CW EPR spectra was 2.0 mW, corresponding to an attenuation of 20 dB on the spectrometer. The reported spectra were obtained by combining several individually recorded scans to ensure that the signal intensity and shape remained constant over time. CW EPR spectra were simulated as absorption powder spectra in MATLAB (R2022b) via full-matrix diagonalization with the pepper function in the EasySpin package. [2-4]

1.5 ^{1}H and ^{31}P NMR Spectroscopy. All NMR spectroscopic measurements were performed on a Bruker AVIII 500 MHz NMR spectrometer at -30 °C unless stated otherwise. Chemical shifts (δ) are reported in parts per million (ppm) and calibrated using deuterated solvent signals for ^{1}H NMR.

1.6 Gas Chromatography. GC-FID chromatograms were recorded on a Shimadzu GC-2030 using a Shimadzu SH-Rtx-5MS (Crossbond 5% diphenyl/95% dimethyl polysiloxane) column (30 m x 0.25 mmID x 0.25 µm df). AOC-30i auto injector used a split ratio of 20:1 with an injection volume of 1 µL and a temperature of 150 °C. The flow rate of carrier gas H_2 was maintained at a constant pressure of 85.2 kPa, and the FID temperature was set to 250 °C. Data analysis was completed through Shimadzu LabSolutions software. All GC yields were obtained through GC calibration curves of the corresponding products with dodecane as an internal standard and the yields were calculated with respect to iron complexes.

1.7 Solution-State Magnetic Susceptibility Measurement. Crystalline material was isolated as reported herein, charged in a scintillation vial with a stir bar and 0.9 mL of d^8-THF to obtain a concentration of 10 mM. Hexamethyldisiloxane (HMDSO) (0.1 mL) was added to the solution and stirred until completely dissolved. Of the solution, 0.6mL was then placed in a J. Young NMR tube. A capillary of HMDSO/d^8-THF (1:1) was then placed into the tube, the tube was sealed, and frozen in liquid nitrogen until run at -30 °C. Measurements were performed on a Bruker AVIII 500 MHz. Calculations were performed in accordance with literature protocol. [5]

1.8 Solid-State Magnetic Susceptibility Measurement. Magnetic susceptibility data were measured from powder samples of solid material in the temperature range 1.8–273 K by using a SQUID magnetometer with a field of 1 T (MPMS-3, Quantum Design). The microcrystalline sample was loaded into the gelatin capsule and restricted with eicosane. All the measurements were done in triplicate. The experimental data were corrected for underlying diamagnetism by use

of tabulated Pascal's constants. [5]

1.9 Cyclic Voltammetry. Cyclic voltammetry (CV) was performed in the glovebox under inert conditions using EMStat4s. Electrodes: Working – glassy carbon. Counter – platinum wire. Pseudo reference – silver wire. Reference – Ag /AgCl (leak-proof). All the peaks were referenced with ferrocene as an external standard.

1.10 Synthetic procedures.

1.10.1 Optimised protocol for preparation of [(dcype)$_2$Fe][BAr^F] (2).

In a nitrogen-filled glovebox, (dcype)FeBr$_2$ (52 mg, 0.08 mmol), dcype (34 mg, 0.08 mmol, 1 eq) and NaBAr^F (71 mg, 0.08 mmol, 1 eq) were dissolved in 3 mL of 2-MeTHF in 14 mL scintillation vial equipped with a stir bar. The solution was cooled to -78 °C in the coldwell (acetone/dry ice) for 10 min. KC$_8$ (25 mg, 0.2 mmol, 2.5 eq) was weighed into a separate vial, and the vial was cooled down to -78 °C. Once cold, the solution of (dcype)FeBr$_2$ was poured onto the KC$_8$ and vigorously mixed for 10 s after which the green solution was quickly filtered through celite to remove unreacted KC$_8$, graphite and KBr. The solution was stirred at -78 °C for 10 min, at which point the colour changed to deep blue and a fine, colourless precipitate (KBr) formed. The reaction was mixed with 3 mL of pentane and filtered through celite. Colourless blocks of the product crystallise overnight at -35°C. The product was washed with cold 2-MeTHF, dried in vacuo, and stored at -35 °C under N$_2$. (Yield: 86 mg, 53%).

^{1}H NMR (500 MHz, d$_8$-Toluene): δ 8.75, 8.13, 3.80, 3.79, 3.77, 3.59, 3.67, 3.55, 2.55, 2.14, 2.13, 1.68, 1.64, 1.55, 1.33, 1.31, 1.29, 1.27, 1.25, 1.22, 1.19, 1.17, 1.15, 0.93, 0.92, 0.90, 0.31 ppm. ^{31}P NMR (202 MHz, d$_8$-Toluene) no resonances. μ_{eff} (SQUID, 243 K) = 4.3(2) μ_B.

Mössbauer parameters (T = 80 K): δ = 0.78 mm/s and $|\Delta E_Q|$ = 0.53 mm/s (solid); δ = 0.78 mm/s $|\Delta E_Q|$ = 0.53 mm/s (frozen 2-MeTHF solution).

1.10.2 Optimised protocol for preparation of (dcype)$_2$Fe (3).

Route 1. In a nitrogen-filled glovebox, (dcype)FeBr$_2$ (52 mg, 0.08 mmol), dcype (34 mg, 0.08 mmol, 1 eq) were dissolved in 3 mL of 2-MeTHF in 14 mL scintillation vial equipped with a stir bar. Excess of Mg powder (97 mg, 4 mmol, 50 equiv.) was added and the reaction was stirred at RT overnight resulting in the colour change from colourless to bright orange. The solvent was reduced to approximately 0.5 ml and the vial was placed in -78 °C coldwell. Bright yellow microcrystalline product is formed after 2 hours at -78°C. The product was washed with cold pentane, dried in vacuo, and stored at -35 °C under N$_2$. (Yield: 30 mg, 42%). Crystals suitable for SC-XRD were grown from saturated pentane solution at RT.

Route 2. In a nitrogen-filled glovebox, [(dcype)$_2$Fe][BAr^F] (**2**) (162 mg, 0.08 mmol) were dissolved in 5 mL of 2-MeTHF in 14 mL scintillation vial equipped with a stir bar. The solution was cooled to -78 °C in the coldwell (acetone/dry ice) for 10 min. KC$_8$ (13 mg, 0.096 mmol, 1.2 equiv) was weighed into a separate vial, and the vial was cooled down to -78 °C. Once cold, the solution of (dcype)FeBr$_2$ was poured onto the KC$_8$ and vigorously mixed for 10 s, the reaction mixture was warmed up to RT and vigorously stirred for 10 min resulting in the colour change from blue to bright orange. The solvent was reduced to approximately 0.5 ml and the vial was placed in -78 °C coldwell. Bright yellow microcrystalline product is formed after 2 hours at -78°C. The product was washed with cold pentane, dried in vacuo, and stored at -35 °C under N$_2$. (Yield: 32 mg, 46%).

μ_{eff} (SQUID, 243 K) = 3.0(2) μ_B.

Mössbauer parameters (T = 80 K): δ = 0.42 mm/s and $|\Delta E_Q|$ = 1.12 mm/s (solid). Due to the poor solubility at lo temperature and decomposition of the compound at higher temperature (Fig. S4) no further solution-state characterisation was pursued.

1.10.3 Reaction of Fe complexes with CyBr. In a nitrogen-filled glovebox, Fe complex (0.03 mmol) was dissolved in 3 mL of THF in a 14 mL scintillation vial equipped with a stir bar. The solution was cooled to 0 °C with a recirculation bath for 10 min. Electrophile (CyBr, 0.3 mmol, 10 equiv.) was weighed into a separate vial, dissolved in 1 mL of THF, and the vial was cooled down to 0 °C. Once cold, the solutions were mixed, and the samples were freeze-trapped at specified timepoints in liquid nitrogen or quenched with 1 M HCl in Et$_2$O and analysed by GC.

1.10.4 Reaction of Fe(I) complexes with (CH$_3$)SiCH$_2$MgCl. In a nitrogen-filled glovebox, Fe(I) complex (0.03 mmol, 61 mg) **2** was dissolved in 3 mL of THF in a 14 mL scintillation vial equipped with a stir bar. The solution was cooled to 0 °C with a recirculation bath for 10 min. Nucleophile ((CH$_3$)SiCH$_2$MgCl, 0.3 mmol, 1 M in diethyl ether, 10 equiv.) was dissolved in 1 mL of THF, and the vial was cooled down to 0 °C. Once cold, the solutions were mixed, and the samples were freeze-trapped in liquid nitrogen or transferred to the J-Young NMR tube for further analysis.

1.10.5 Single turnover experiment. In a nitrogen-filled glovebox, Fe(I) complex (0.03 mmol, 61 mg) **2** was dissolved in 0.3 mL of THF in a 14 mL scintillation vial equipped with a stir bar. The solution was cooled to 0 °C with a recirculation bath for 10 min. Electrophile (CyBr, 0.6 mmol, 20 equiv.) was weighed into a separate vial and added to the solution of **2**. Nucleophile (PhMgBr, 0.03 mmol, 0.92 M in THF, 1 equiv.) was added dropwise over the course of 10 min. Once the addition was complete, the reaction mixture was warmed to RT and stirred for 30 min, after which the samples were freeze-trapped in liquid nitrogen or quenched with 1 M HCl in Et_2O and analysed by GC.

2. Cyclic Voltammetry (CV)

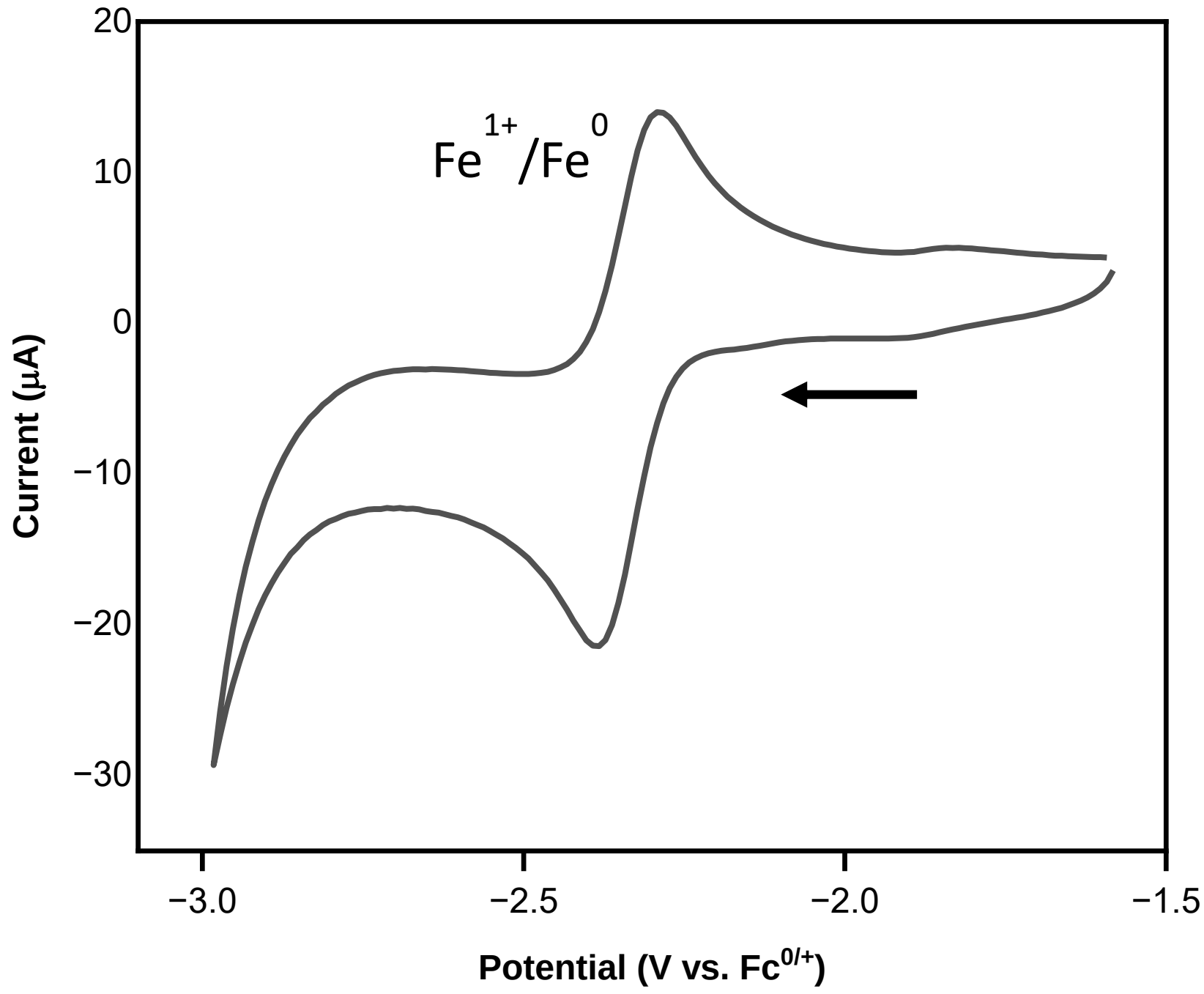

Figure S1. Cyclic voltammograms of **2** (5 mM) in 0.1 M [^{n}Bu$_4$N][PF$_6$] in THF at room temperature (approximately 25 °C). Scan rate: 0.025 V/s.

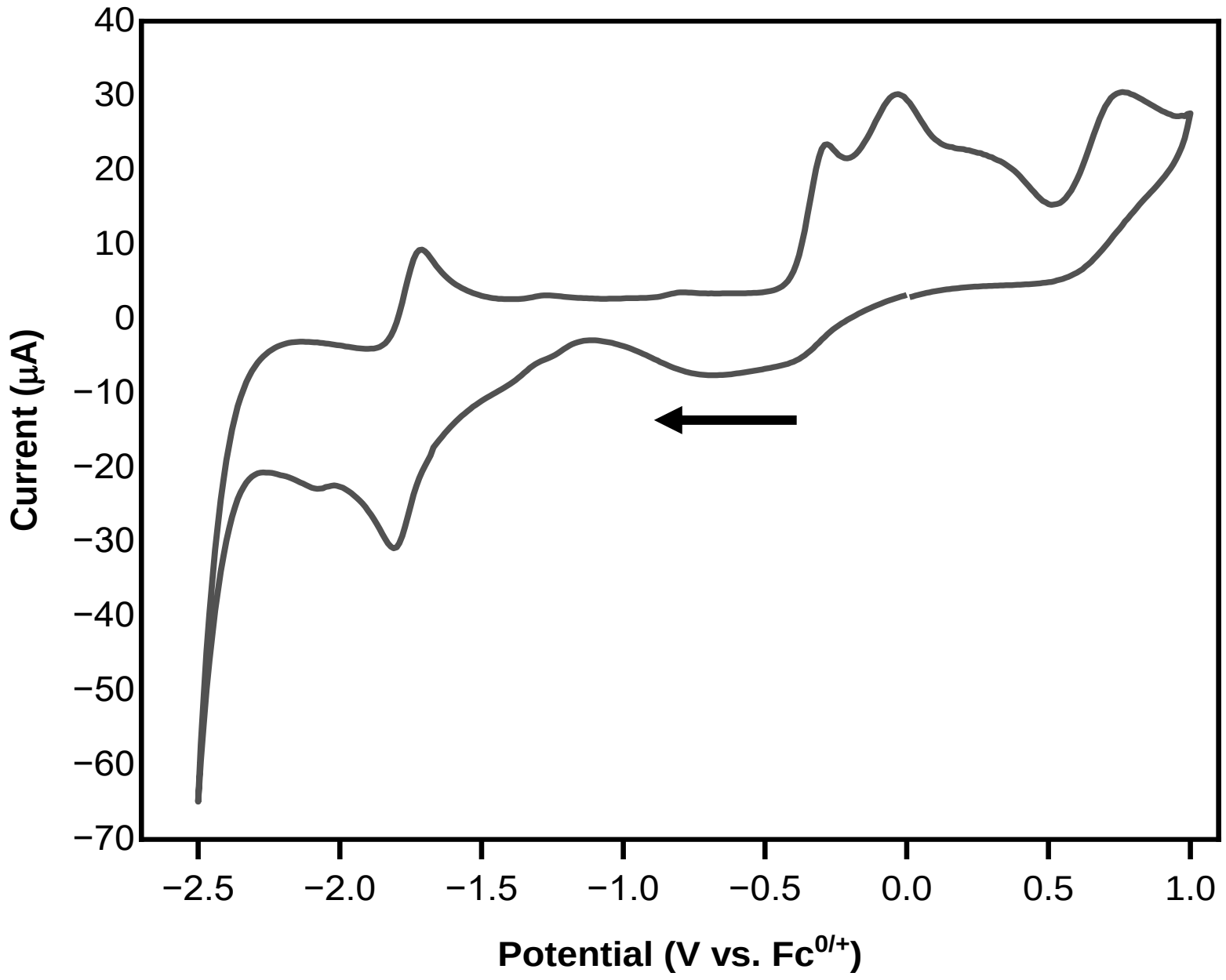

Figure S2. Full cyclic voltammograms of **2** (5 mM) in 0.1 M [^{n}Bu$_4$N][PF$_6$] in THF at room temperature (approximately 25 °C). Scan rate: 0.025 V/s. Note the absence of reversible redox events besides Fe^{1+}/Fe^{0} couple.

3. **Supplementary ^{57}Fe Mössbauer spectroscopy**

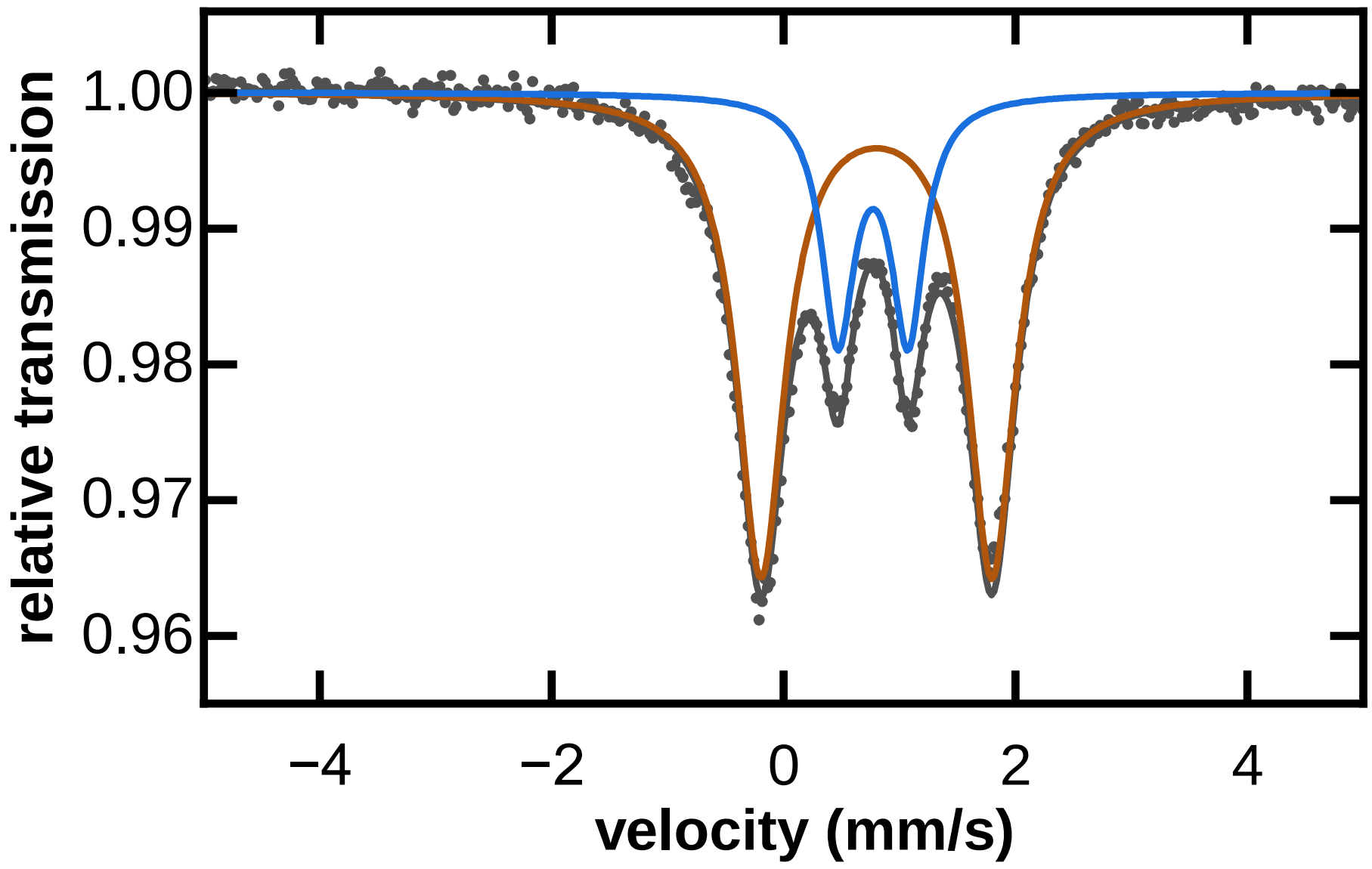

Figure S3. Freeze-trapped ^{57}Fe Mössbauer spectrum of the reaction between **1** and 2.5 equiv. of KC_8 at -78 °C in the presence of extra equvalent of dcype and 1 equiv. of $NaBAr^F_4$ after 30 s. Mössbauer parameters for brown component (77%) δ = 0.79 mm/s $|\Delta E_Q|$ = 1.99 mm/s ([(dcype)Fe(μ-Br)]),[6] blue component (23 %) δ = 0.78 mm/s $|\Delta E_Q|$ = 0.53 mm/s **2**.

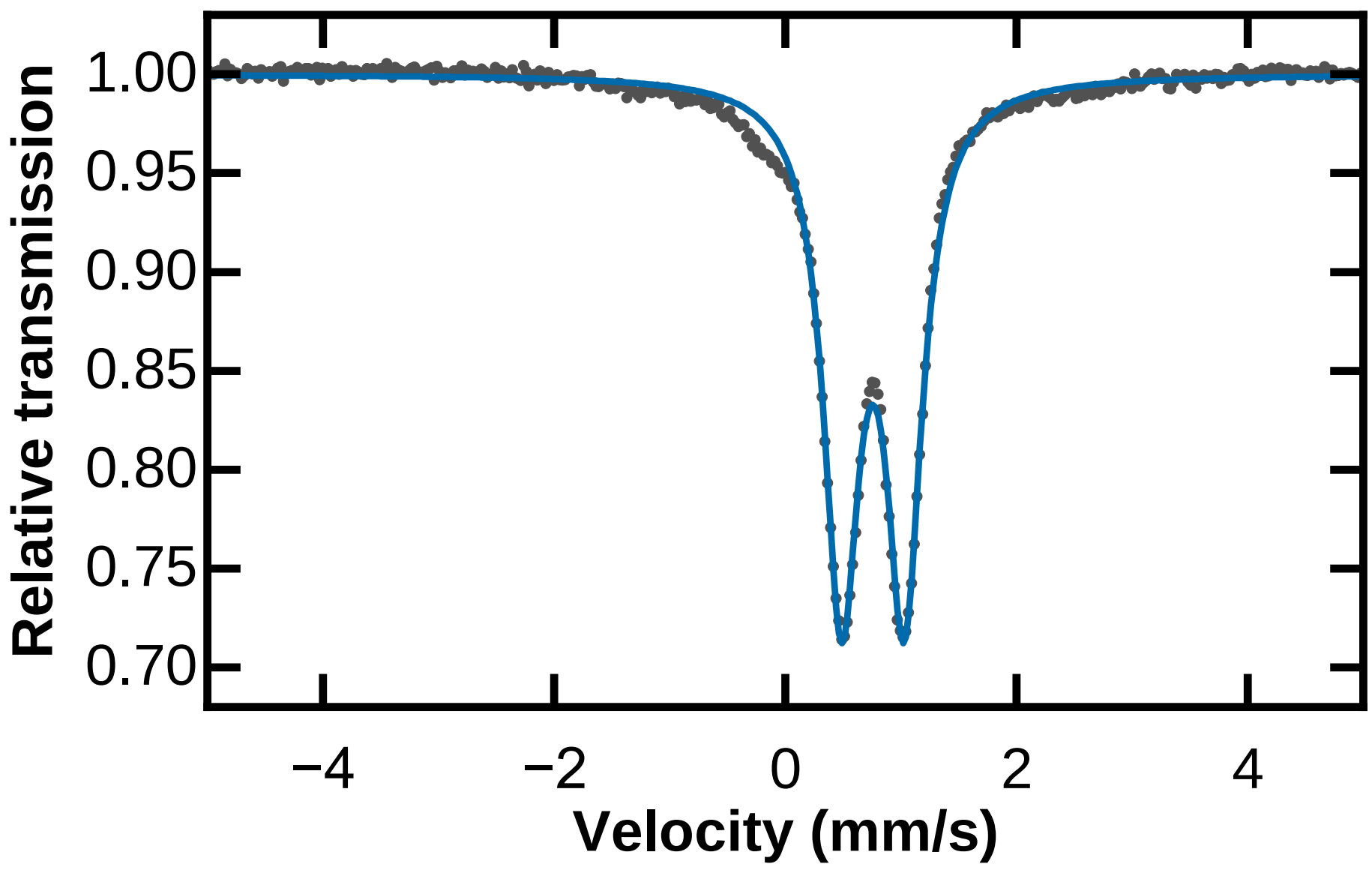

Figure S4. Freeze-trapped ^{57}Fe Mössbauer spectrum of the reaction between **1** and 2.5 equiv. of KC_8 at -78 °C in the presence of extra equvalent of dcype and 1 equiv. of $NaBAr^F_4$ after filtering through celite and incubating the reaction mixture for 10 min at -78 °C. Mössbauer parameters for blue component δ = 0.78 mm/s $|\Delta E_Q|$ = 0.53 mm/s **2**.

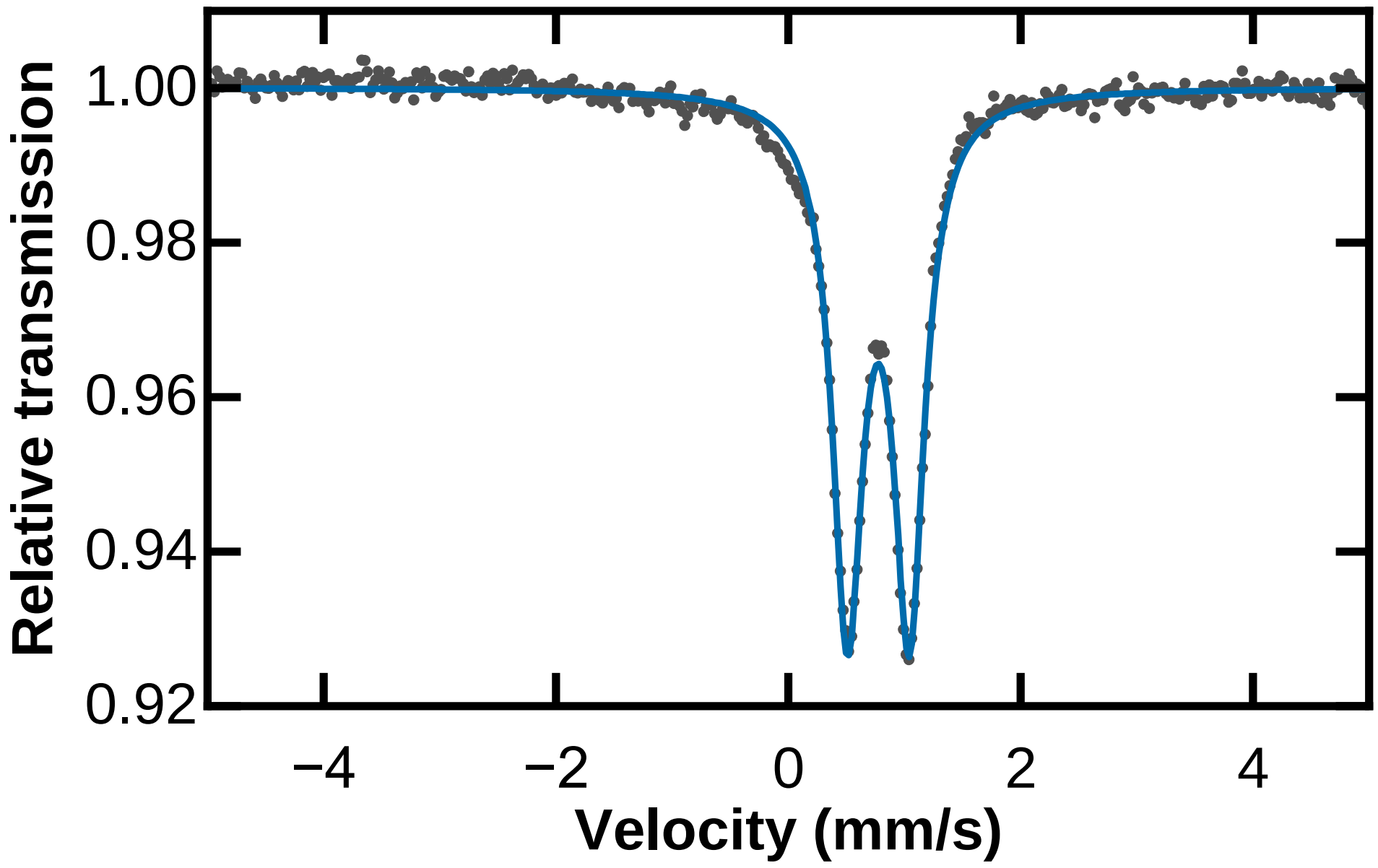

Figure S5. 80 K frozen 2-MeTHF solution ^{57}Fe Mössbauer spectrum of **2**, characterised by a doublet with δ = 0.78 mm/s and $|\Delta E_Q|$ = 0.53 mm/s.

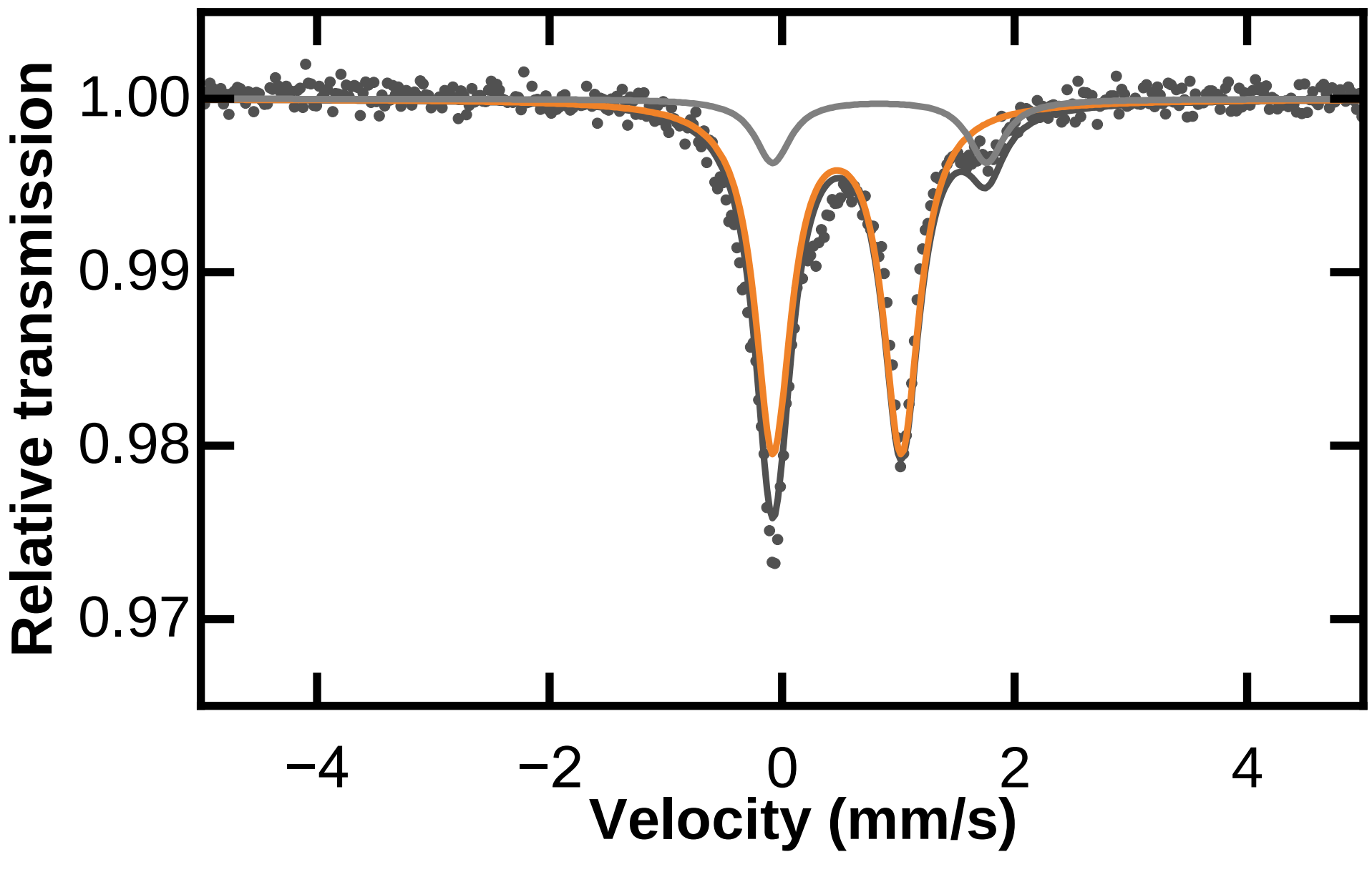

Figure S6. Freeze-trapped ^{57}Fe Mössbauer spectrum of the reaction between **1** and 50 equiv. of Mg powder at RT overnight. Mössbauer parameters for orange component (85%) with δ = 0.42 mm/s and $|\Delta E_Q|$ = 1.12 mm/s, grey component (15%) δ = 0.79 mm/s and $|\Delta E_Q|$ = 1.84 mm/s.

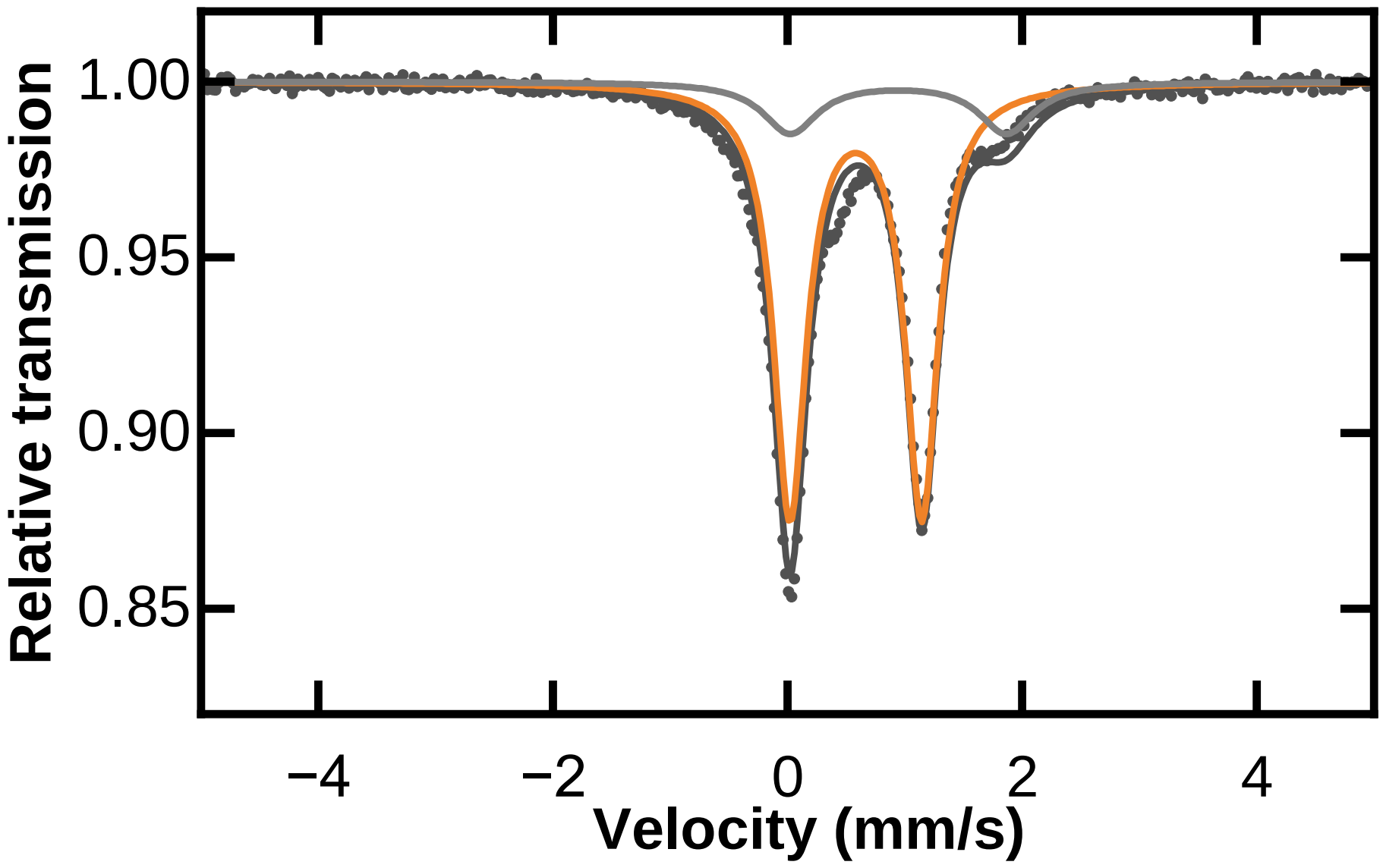

Figure S7. 80 K frozen ^{57}Fe Mössbauer spectrum of solid **3**, characterised by a doublet with δ = 0.42 mm/s and $|\Delta E_Q|$ = 1.12 mm/s (orange component, 83%). Note the presence of an identified decomposition product with δ = 0.79 mm/s and $|\Delta E_Q|$ = 1.84 mm/s (grey component, 17%)

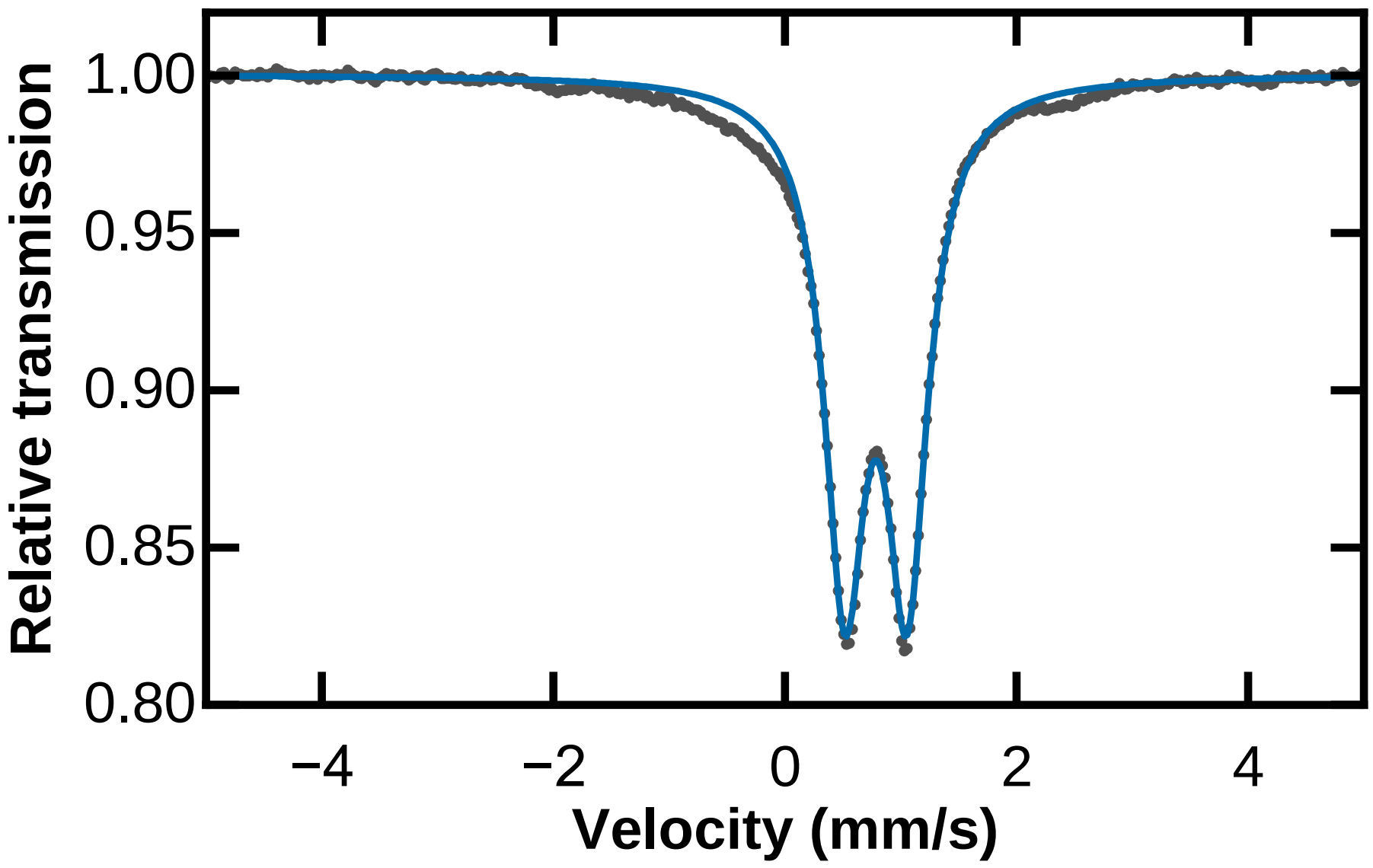

Figure S8. Freeze-trapped ^{57}Fe Mössbauer spectrum of the reaction between **2** and 1 equiv. of PhMgBr at -78 °C for 5 min in THF. No reaction is observed. Mössbauer parameters for blue component δ = 0.78 mm/s $|\Delta E_Q|$ = 0.53 mm/s **2**.

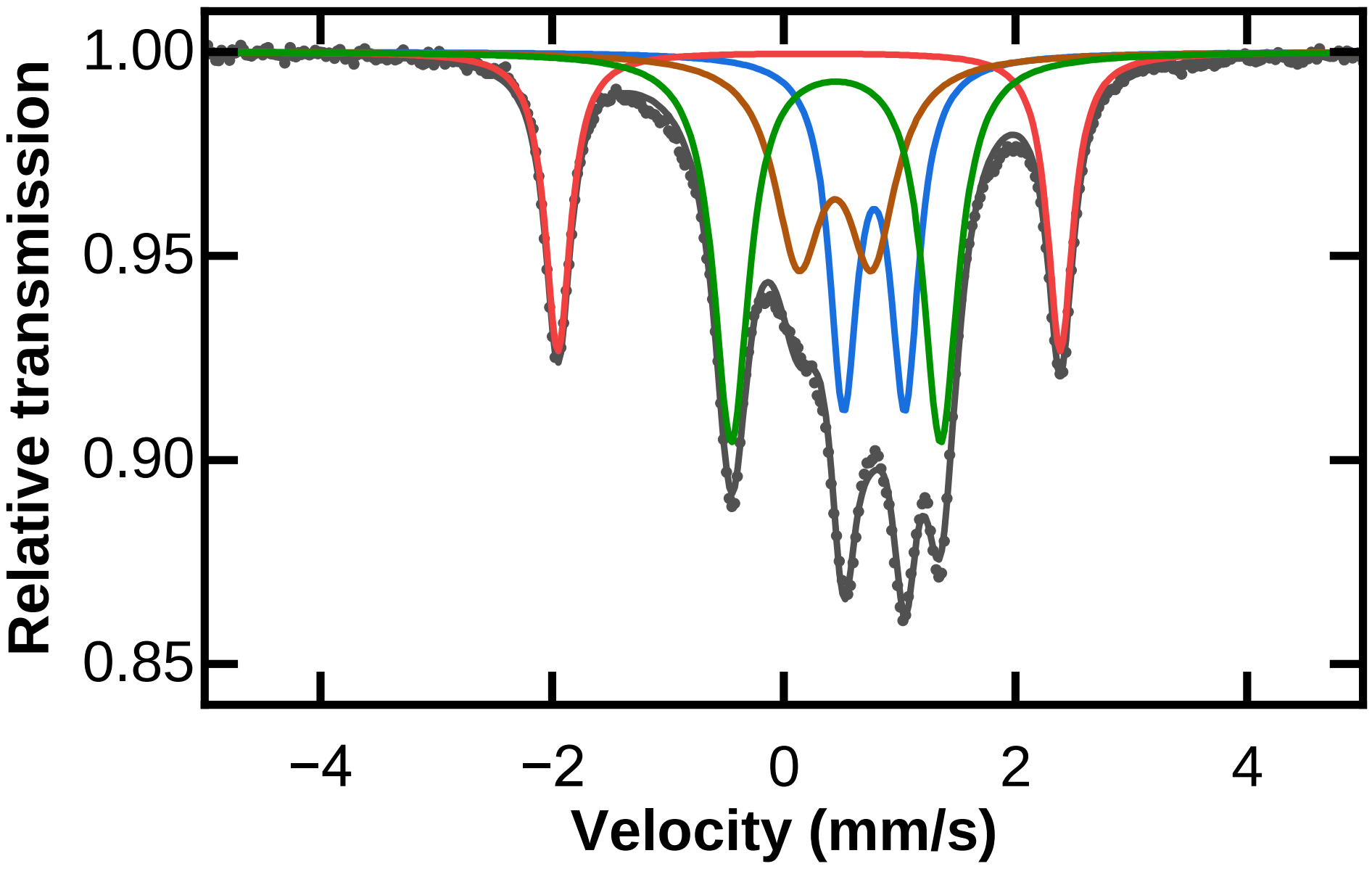

Figure S9. Freeze-trapped ^{57}Fe Mössbauer spectrum of the reaction between **2** and 1 equiv. of PhMgBr at 0 °C for 5 min in THF. Note the formation of multiple species consistent with decomposition. Mössbauer parameters: blue component δ = 0.78 mm/s $|\Delta E_Q|$ = 0.53 mm/s **2** (24%); red component δ = 0.22 mm/s $|\Delta E_Q|$ = 4.34 mm/s (19%); green component δ = 0.45 mm/s $|\Delta E_Q|$ = 1.80 mm/s (34%); brown component δ = 0.44 mm/s $|\Delta E_Q|$ = 0.64 mm/s (23%).

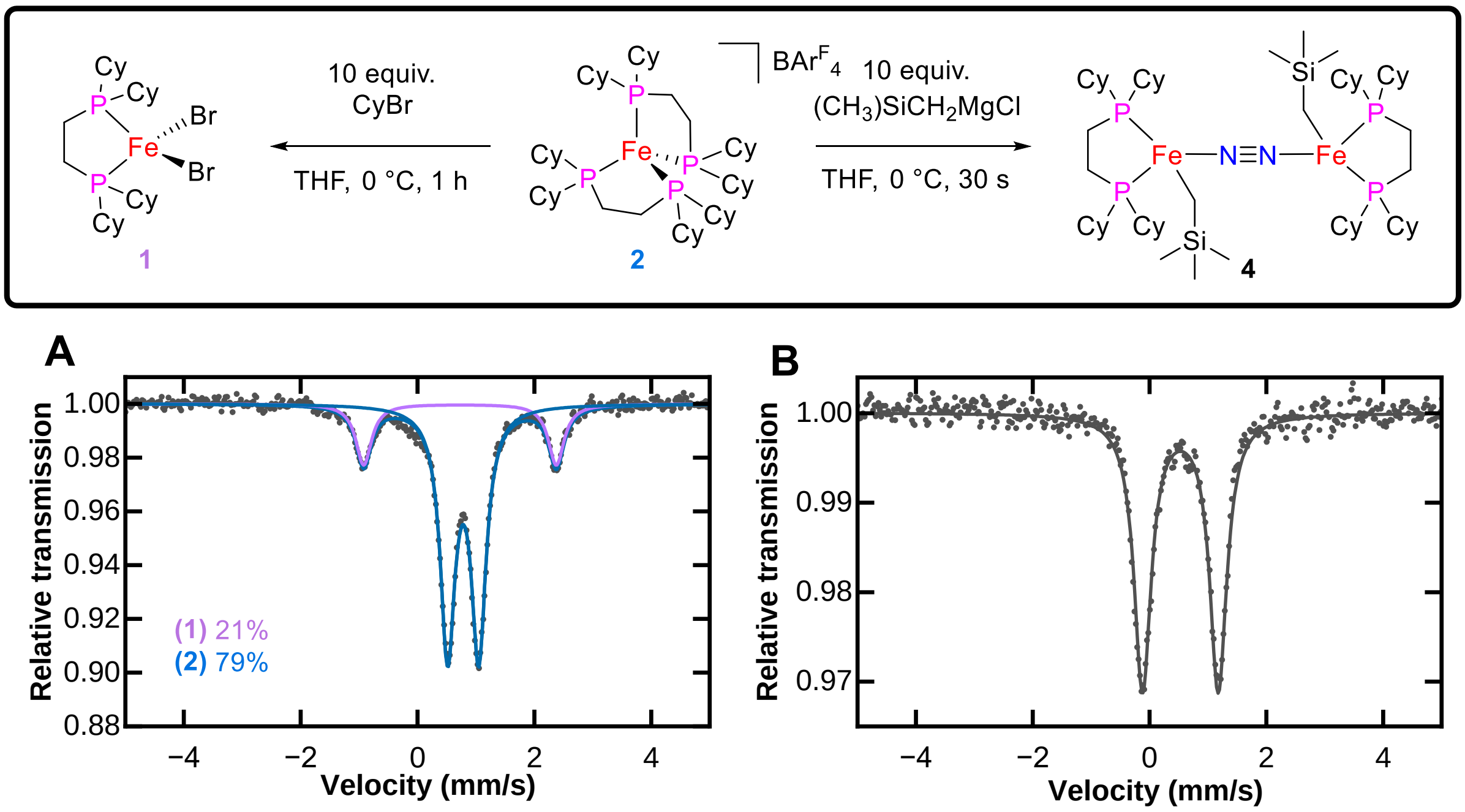

Figure S10. Reactivity of [(dcype)$_2$Fe][BAr^F] **2**. (A) Freeze-trapped ^{57}Fe Mössbauer spectrum of the reaction between **2** and 10 equiv. of CyBr after 1 h at 0 °C. Note that the minimal consumption of **2** indicates limited reactivity towards the electrophile. (B) Freeze-trapped ^{57}Fe Mössbauer spectrum of the reaction between **2** and 10 equiv. of $(CH_3)SiCH_2MgCl$ after 30 s at 0 °C. Mössbauer parameters for purple component δ = 0.73 mm/s $|\Delta E_Q|$ = 3.20 mm/s **1**, blue component δ = 0.78 mm/s $|\Delta E_Q|$ = 0.53 mm/s **2**, black component δ = 0.51 mm/s $|\Delta E_Q|$ = 1.33 mm/s **4**.

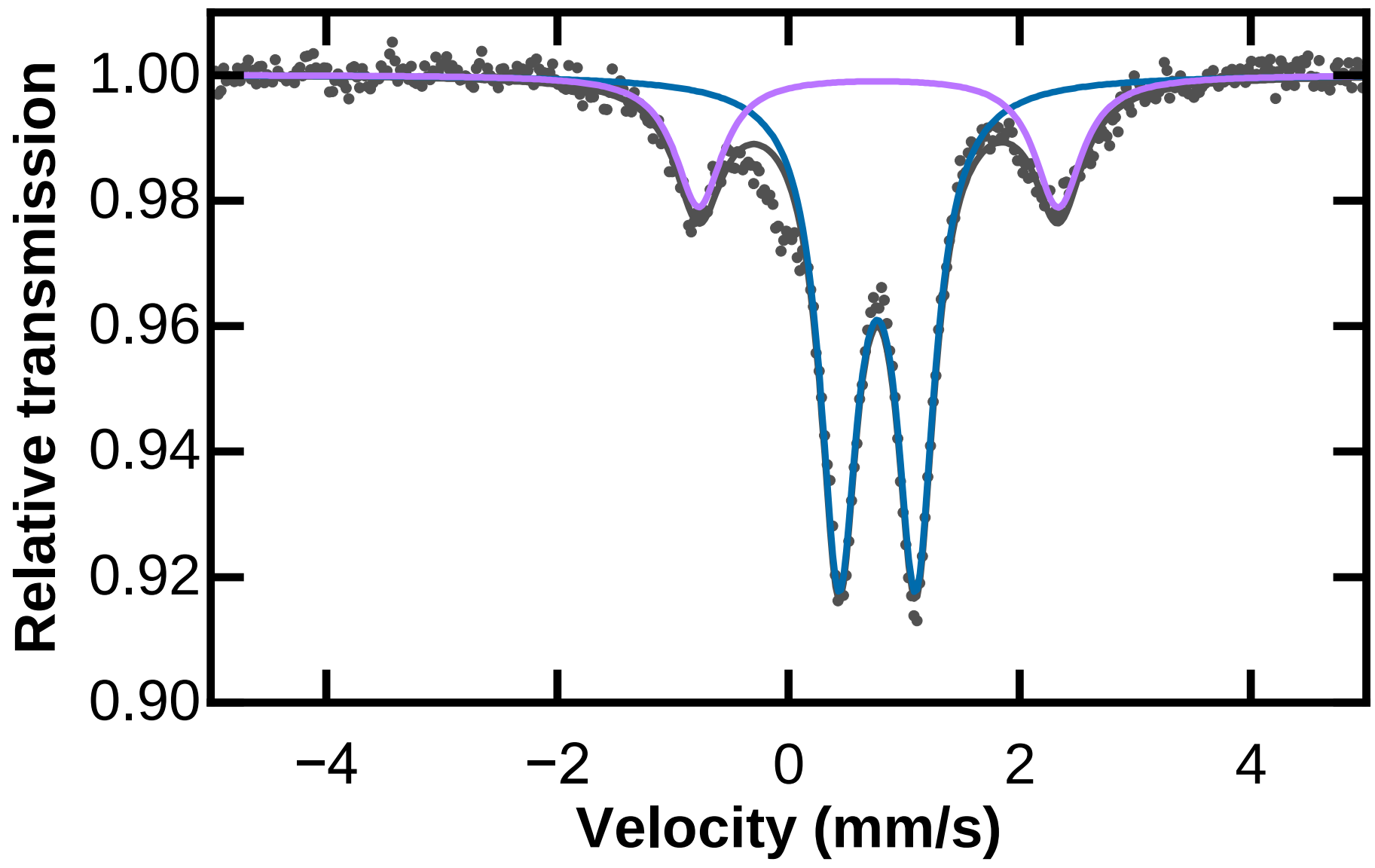

Figure S11. Freeze-trapped ^{57}Fe Mössbauer spectrum of the reaction between **2** and 10 equiv. of CyBr in the presence of excess ligand (5 equiv. dcype) after 1 h at 0 °C. Note that the minimal consumption of **2** indicates limited reactivity towards the electrophile. Mössbauer parameters for purple component (77%) δ = 0.73 mm/s $|\Delta E_Q|$ = 3.20 mm/s **1**, blue component (23%) δ = 0.78 mm/s $|\Delta E_Q|$ = 0.53 mm/s **2**.

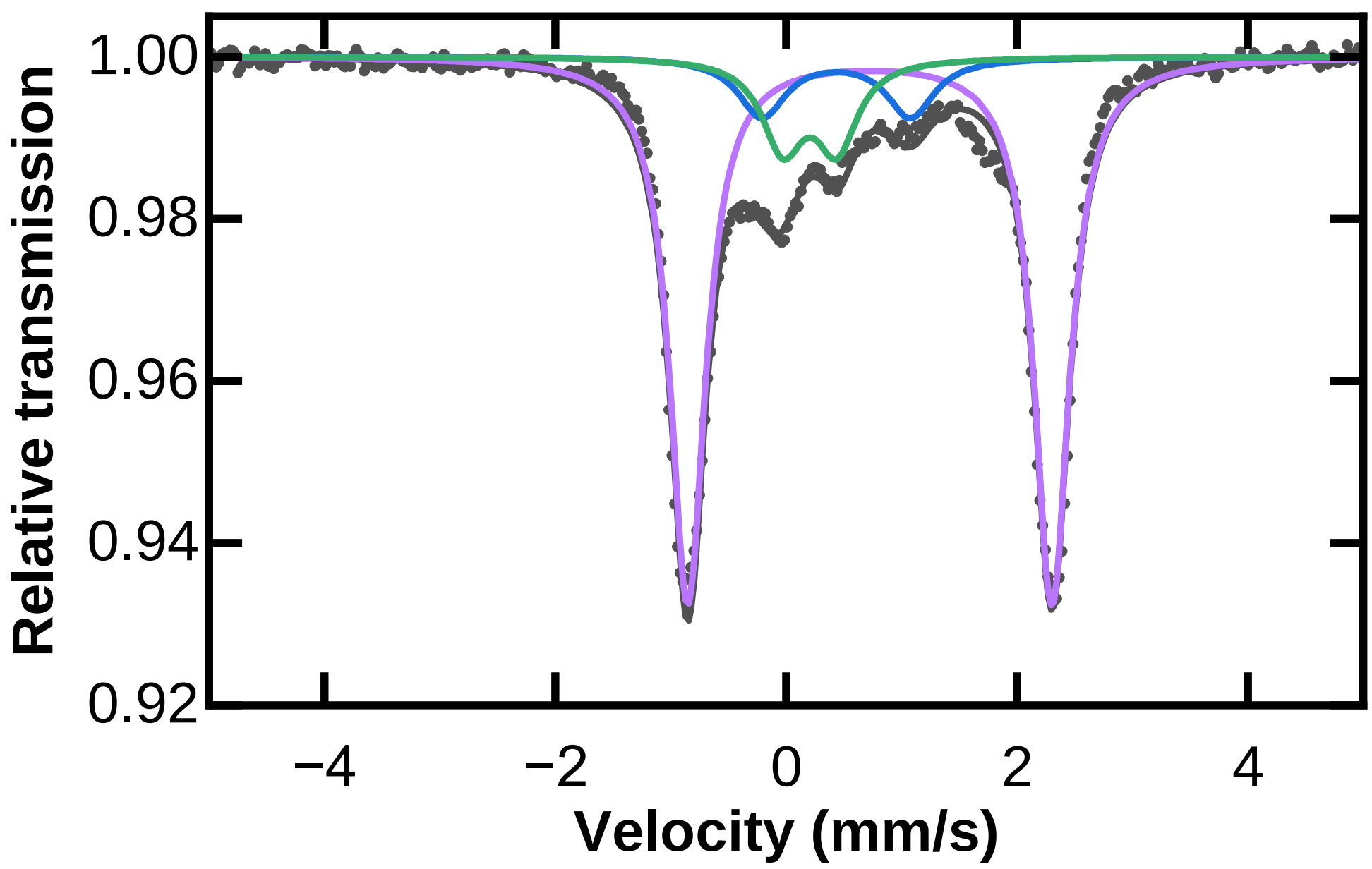

Figure S12. Freeze-trapped ^{57}Fe Mössbauer spectrum of the reaction between **3** and 10 equiv. of CyBr at 0 °C after 30 s in THF. Mössbauer parameters: purple component δ = 0.73 mm/s $|\Delta E_Q|$ = 3.20 mm/s (**1**, 81%); blue component δ = 0.43 mm/s $|\Delta E_Q|$ = 1.28 mm/s (9%); green component δ = 0.20 mm/s $|\Delta E_Q|$ = 0.47 mm/s (10%).

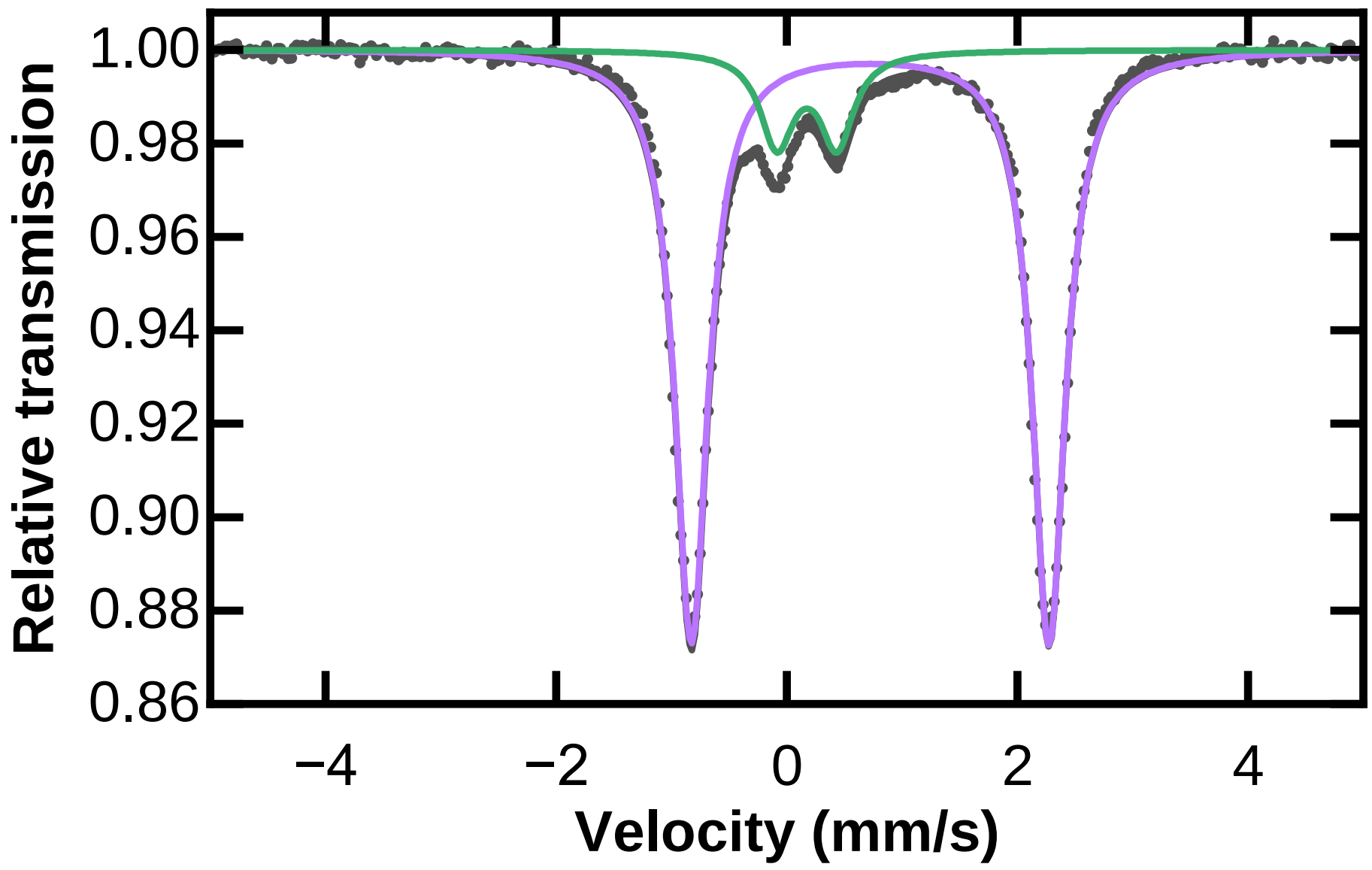

Figure S13. Freeze-trapped ^{57}Fe Mössbauer spectrum of the reaction between **3** and 10 equiv. of CyBr in the presence of excess ligand (5 equiv. dcype) at 0 °C after 30 s in THF. Mössbauer parameters: purple component δ = 0.73 mm/s $|\Delta E_Q|$ = 3.20 mm/s (**1**, 86%); green component δ = 0.20 mm/s $|\Delta E_Q|$ = 0.47 mm/s (14%).

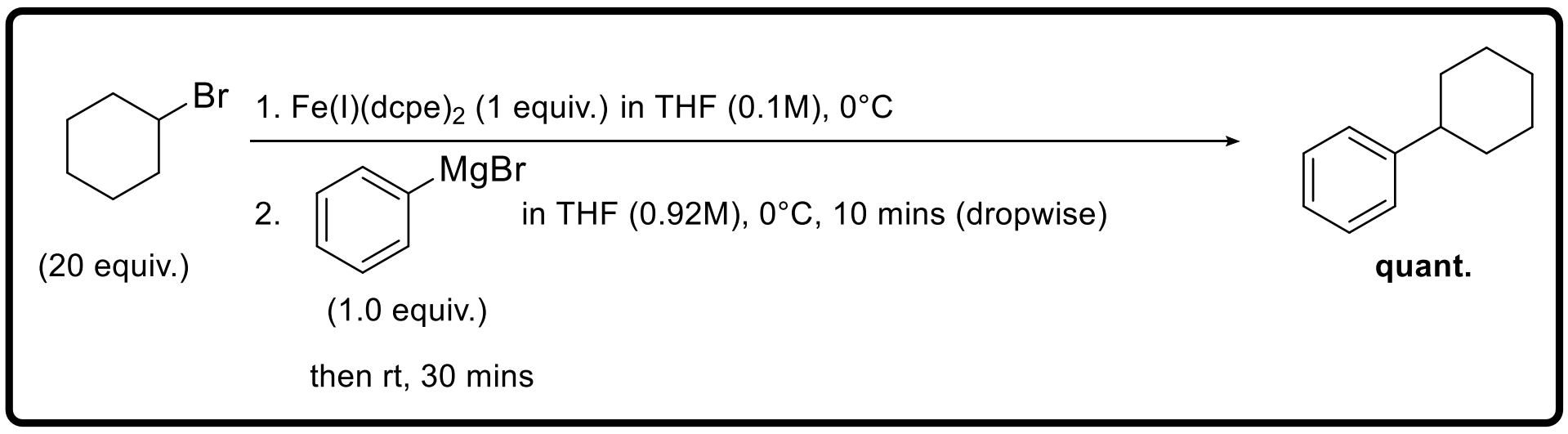

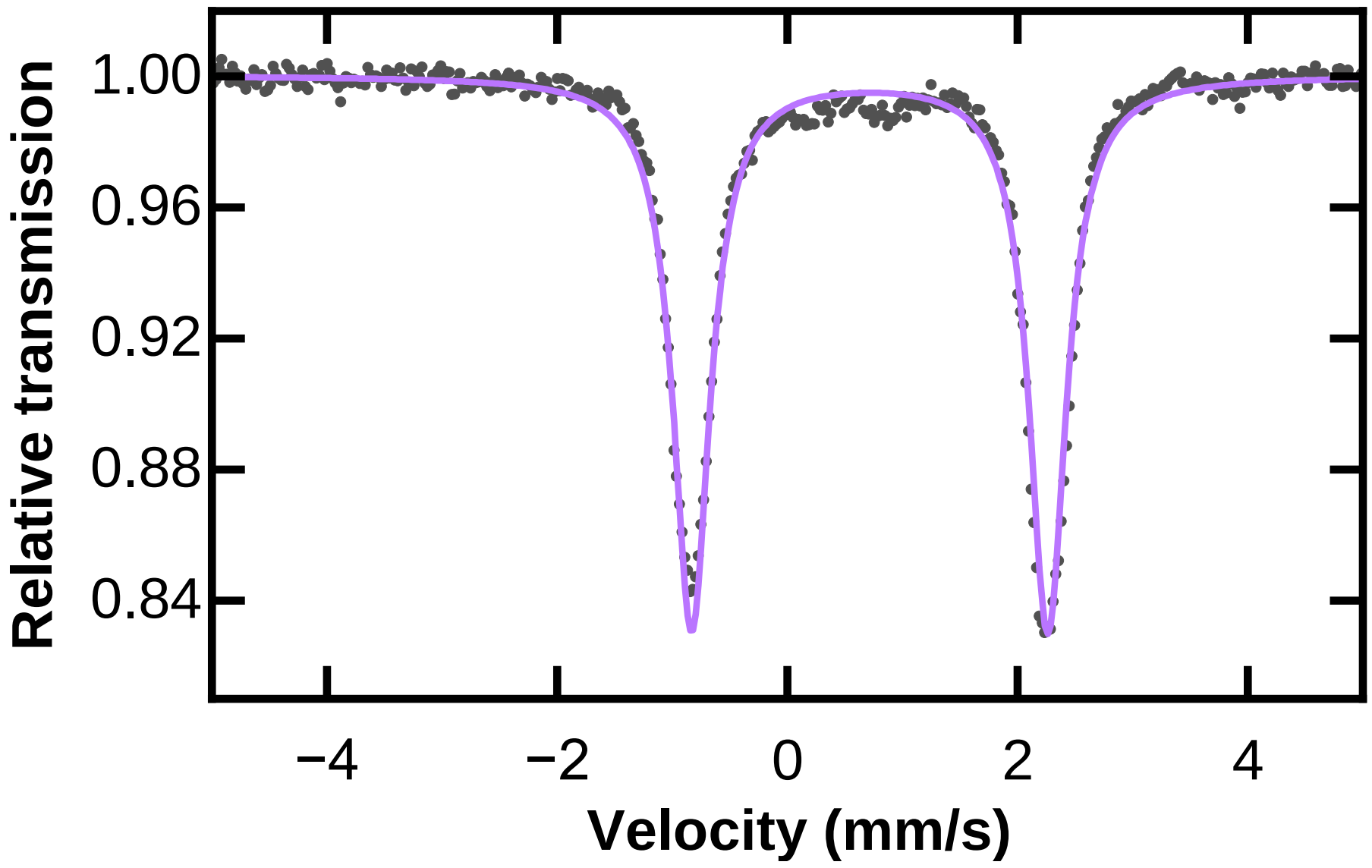

Figure S14. Single-turnover experiment of [(dcype)$_2$Fe][BAr^F] **2**. Freeze-trapped ^{57}Fe Mössbauer spectrum of the reaction between **2**, 20 equiv. of CyBr and 1 equiv. of PhMgBr. The quantitative formation of the organic product is observed by ^1H NMR (CH_2Br_2 was used as internal standard). Mössbauer parameters for purple component δ = 0.73 mm/s $|\Delta E_Q|$ = 3.20 mm/s **1**.

4. **Supplementary Continuous Wave (CW) X-band EPR spectroscopy**

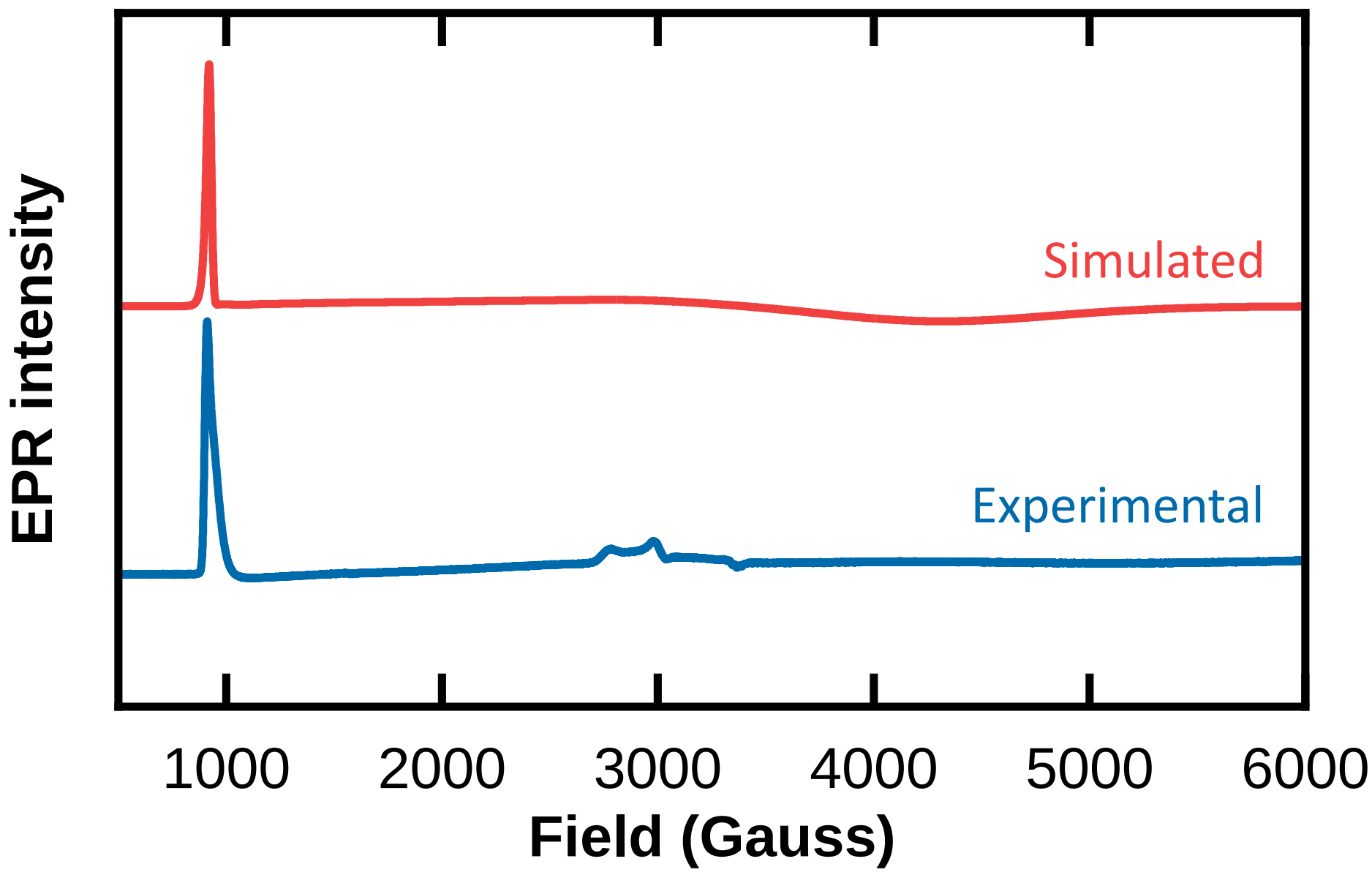

Figure S15. 10 K EPR spectrum of the 2-MeTHF frozen solution of **2**. The spectrum was simulated with S = 1/2 Hamiltonian with parameters: g_1 = 7.23, g_2 = 1.01 g_3 = 1.01.

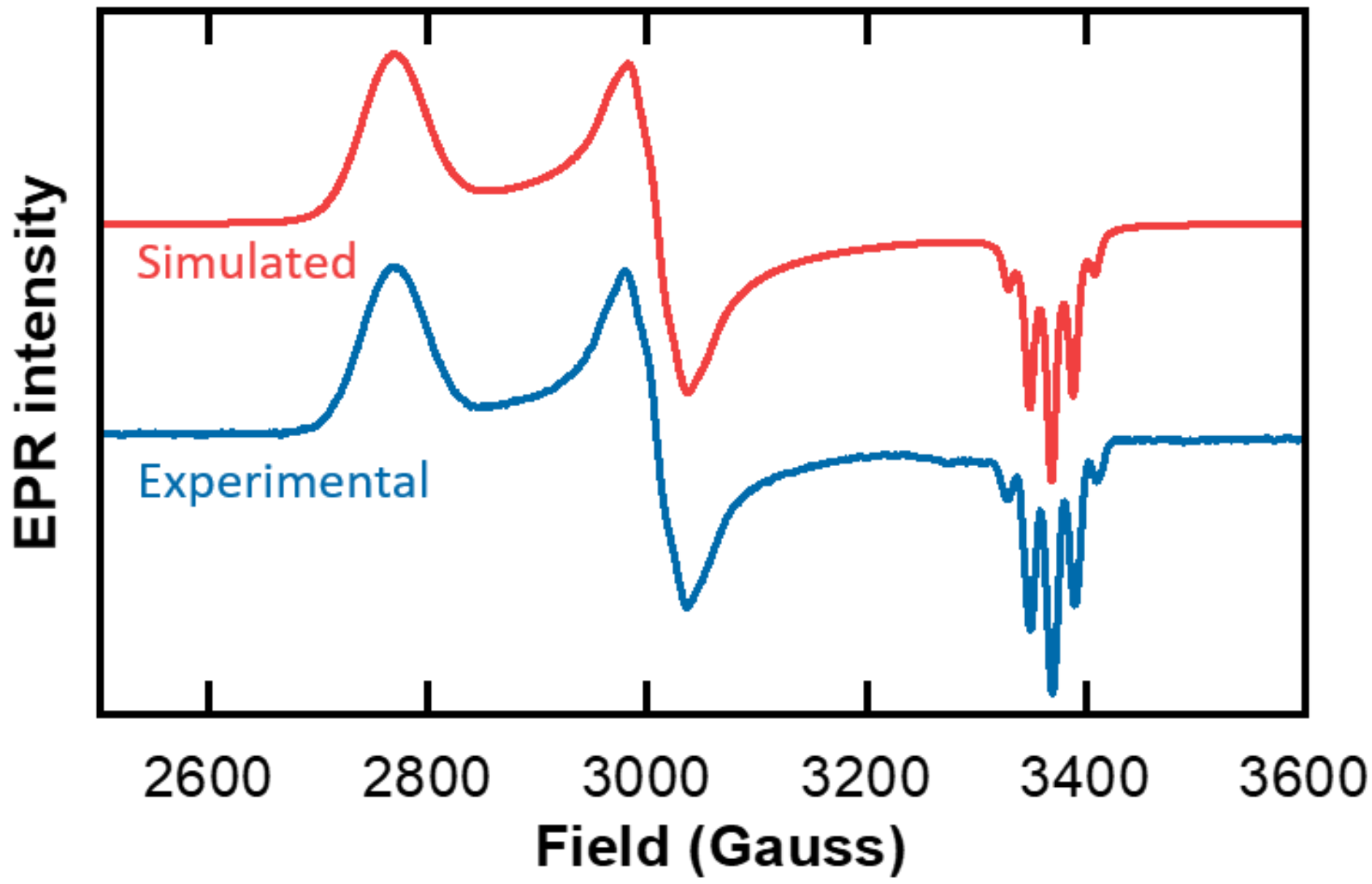

Figure S16. Enlarged 10 K EPR spectrum of **2** in 2-MeTHF glass showing S = 1/2 species corresponding to the square planar [(dcype)$_2$Fe][BAr^F] complex. The spectrum was simulated with g values: g_1 = 2.42, g_2 = 2.23 g_3 = 1.99, and the hyperfine coupling to four equivalent ^{31}P with constant: A_2 = 55.4 and A_3 = 55.5 MHz.

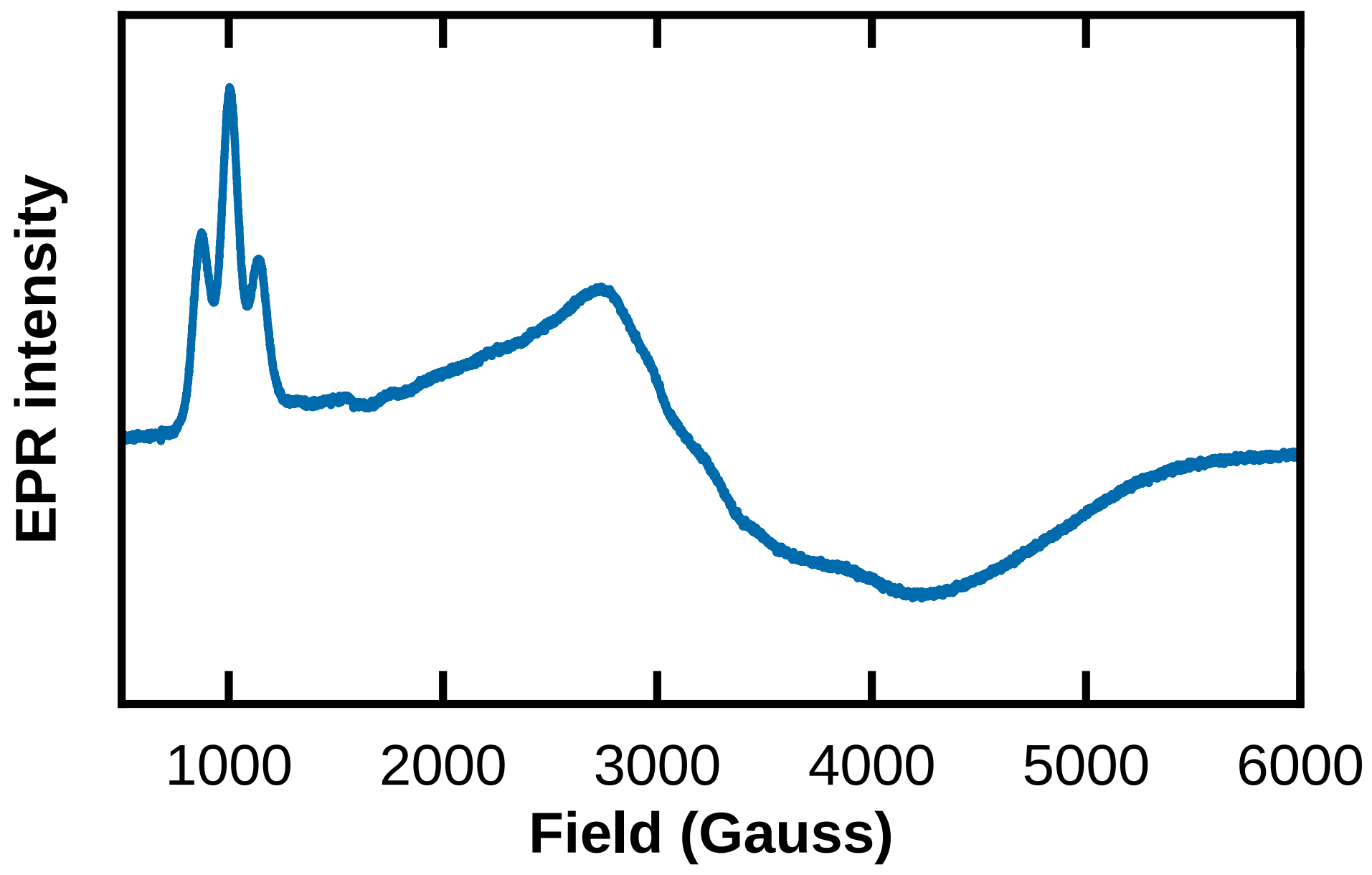

Figure S17. 10 K EPR spectrum of solid **2**.

5. Supplementary NMR spectroscopy

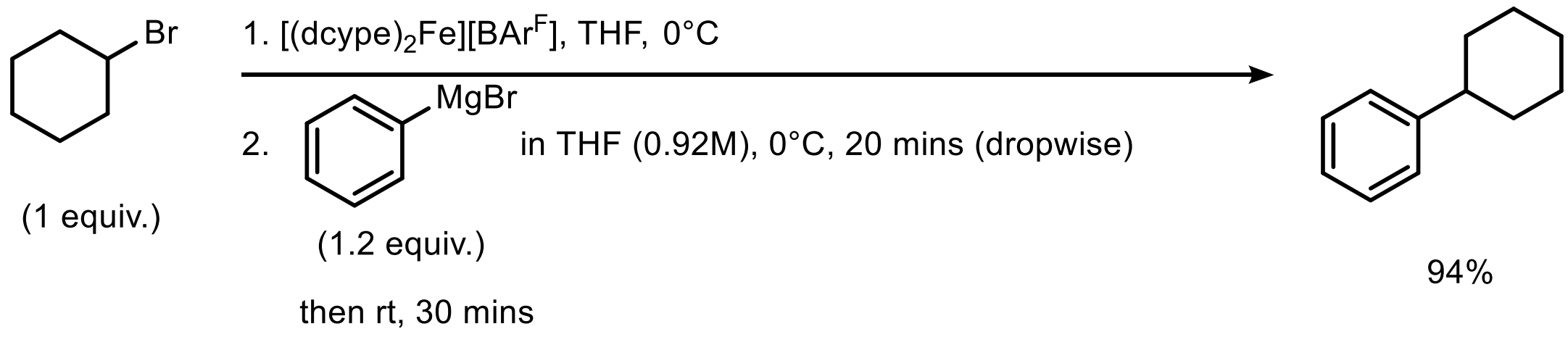

Scheme S1. Kumada cross-coulling reaction between CyBr and PhMgBr catalysed by [(dcype)2Fe][BArF]. The formation of the product (96%) is observed by ^{1}H NMR (CH_2Br_2 was used as internal standard).

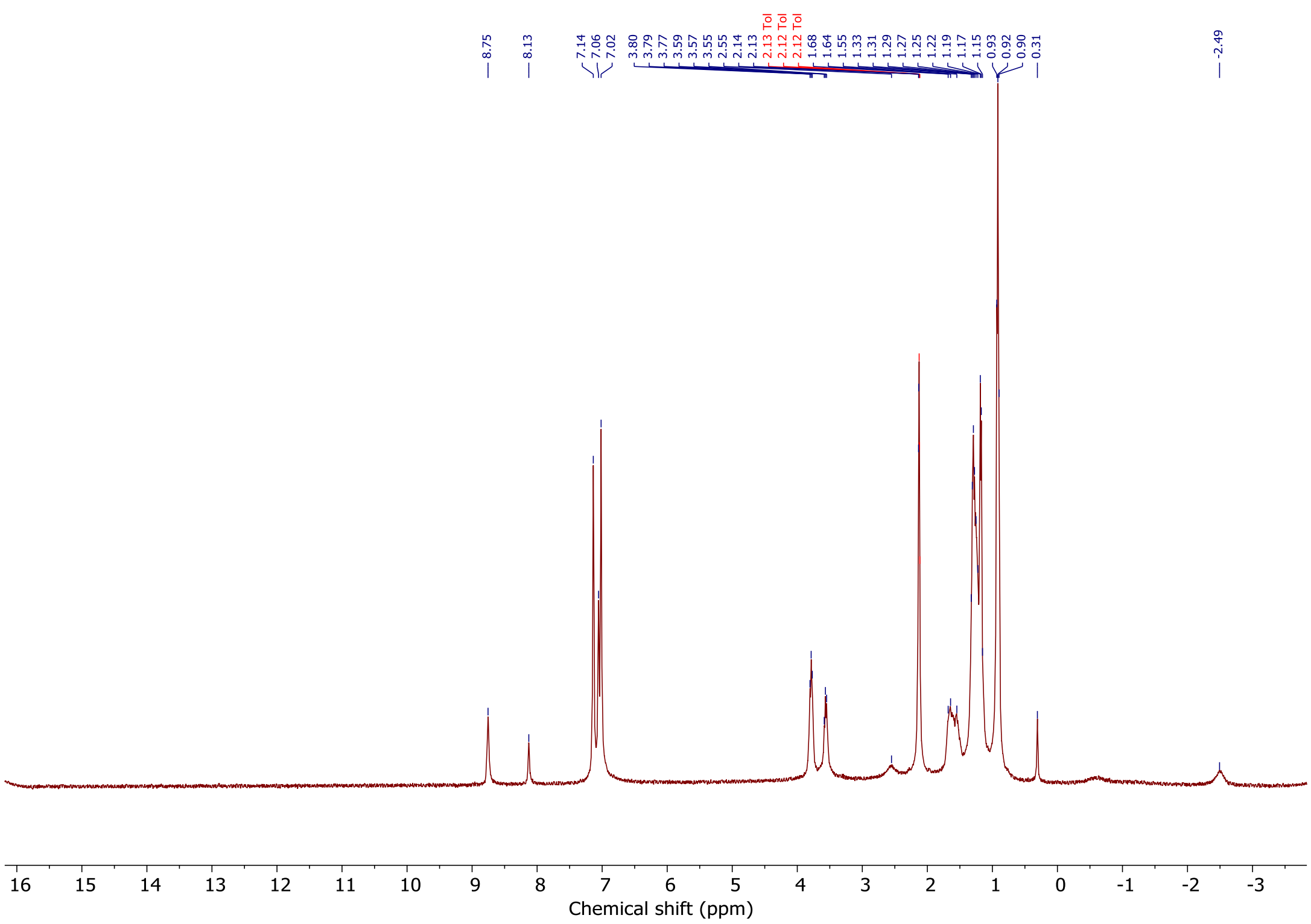

Figure S18. ^{1}H NMR (500 MHz) spectrum of [(dcype)$_2$Fe][BAr^F] **2** in d^8-Toluene.

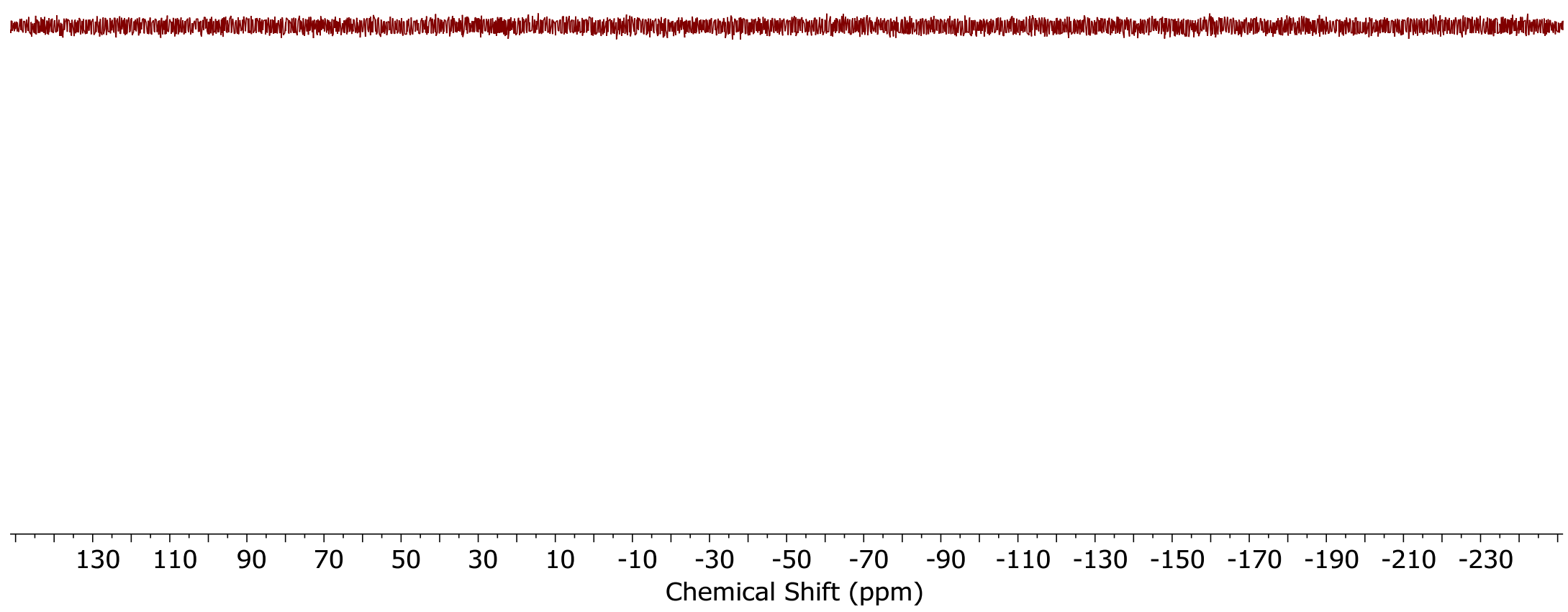

Figure S19. ^{31}P {^{1}H} NMR (202 MHz) spectrum of [(dcype)$_2$Fe][BAr^F] **2** in d^8-Toluene

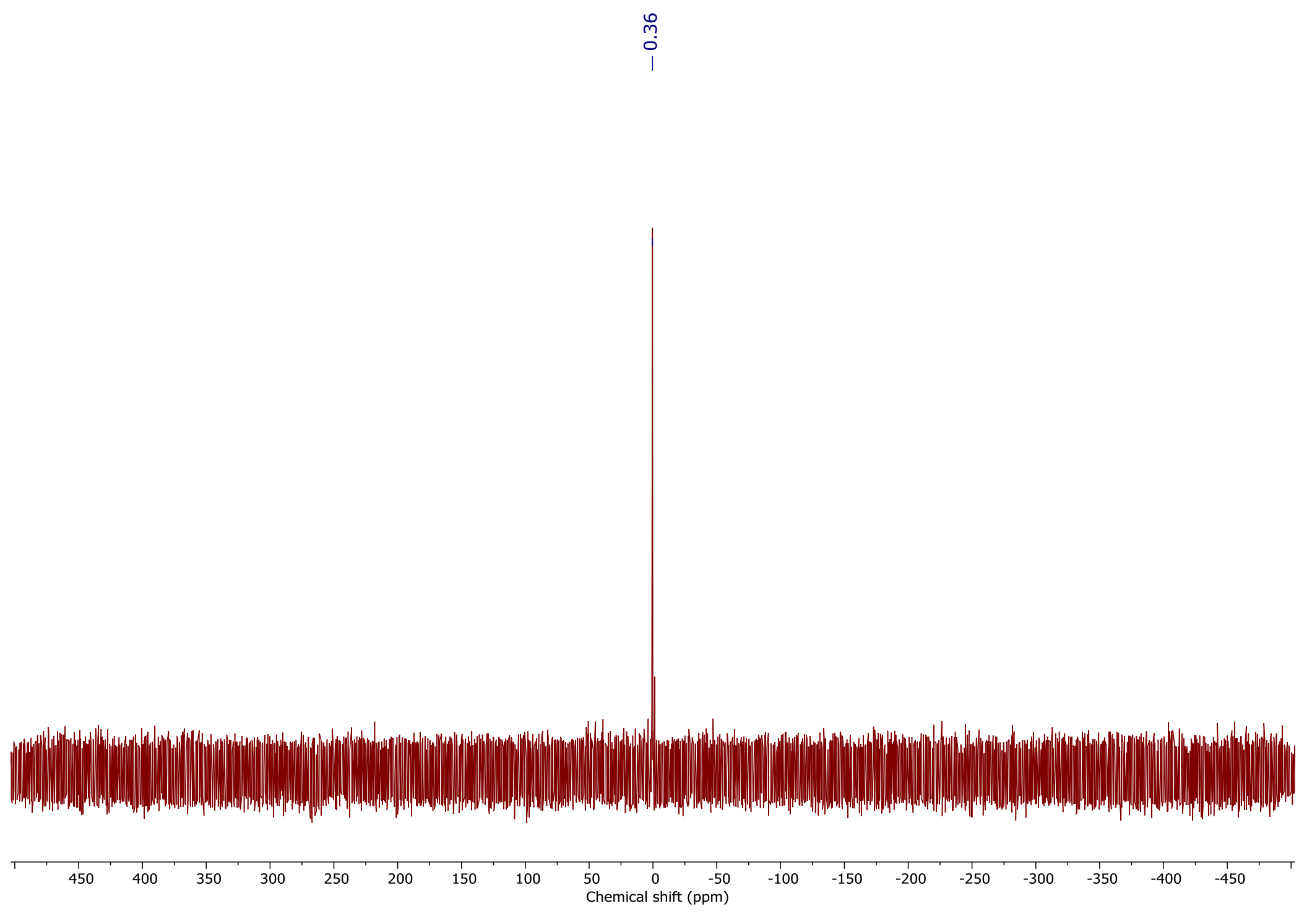

Figure S20. ^{31}P {^{1}H} NMR (202 MHz) spectrum of the reaction of **2** with 10 equiv. of $(CH_3)SiCH_2MgCl$ in d^8-THF. The signal with δ = 0.36 corresponds to the free dcype.

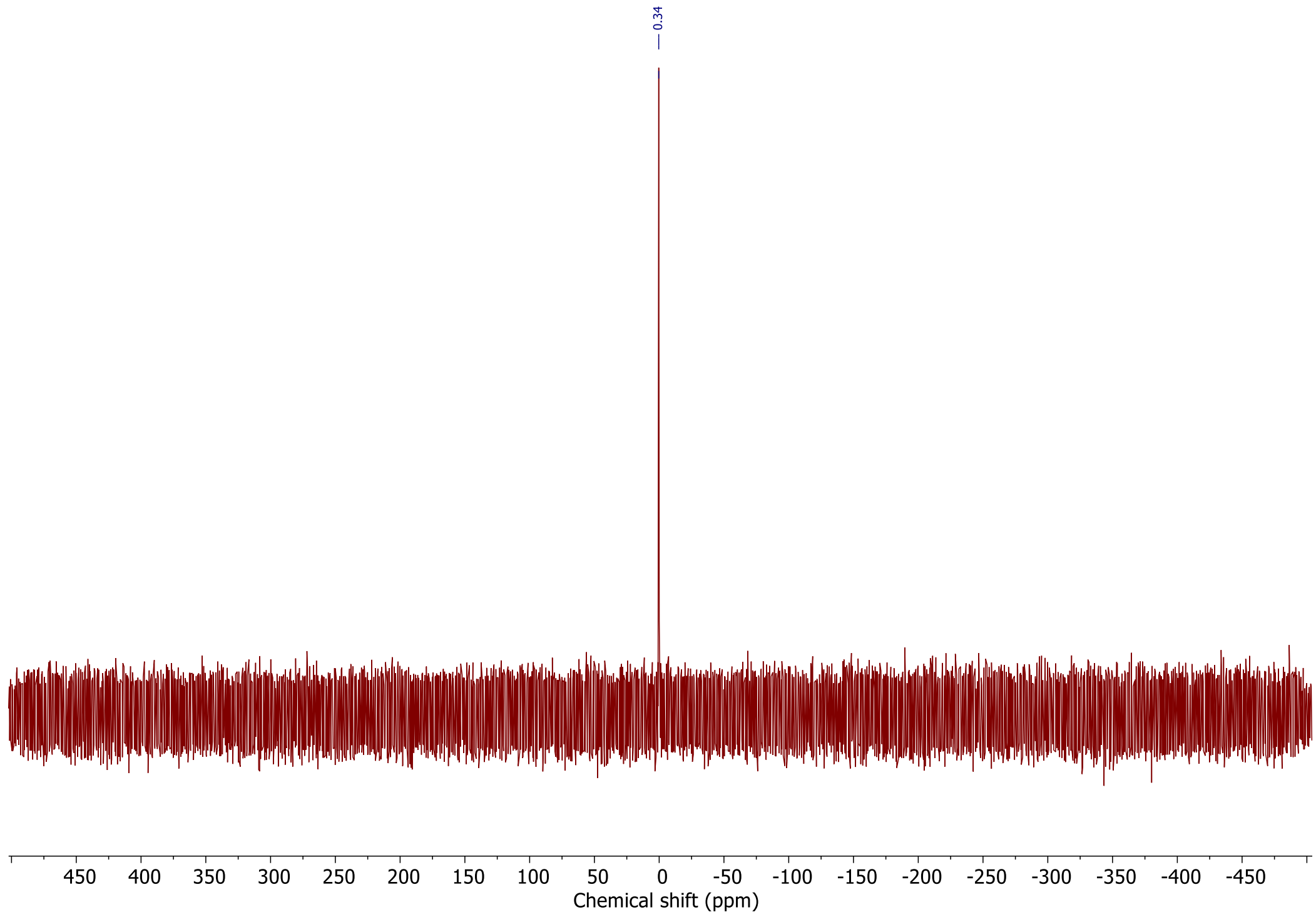

Figure S21. $^{31}P\{^{1}H\}$ NMR (202 MHz) spectrum of the reaction of **3** with 10 equiv. of CyBr in d^8-THF. The signal with δ = 0.36 corresponds to the free dcype.

6. Magnetometry data

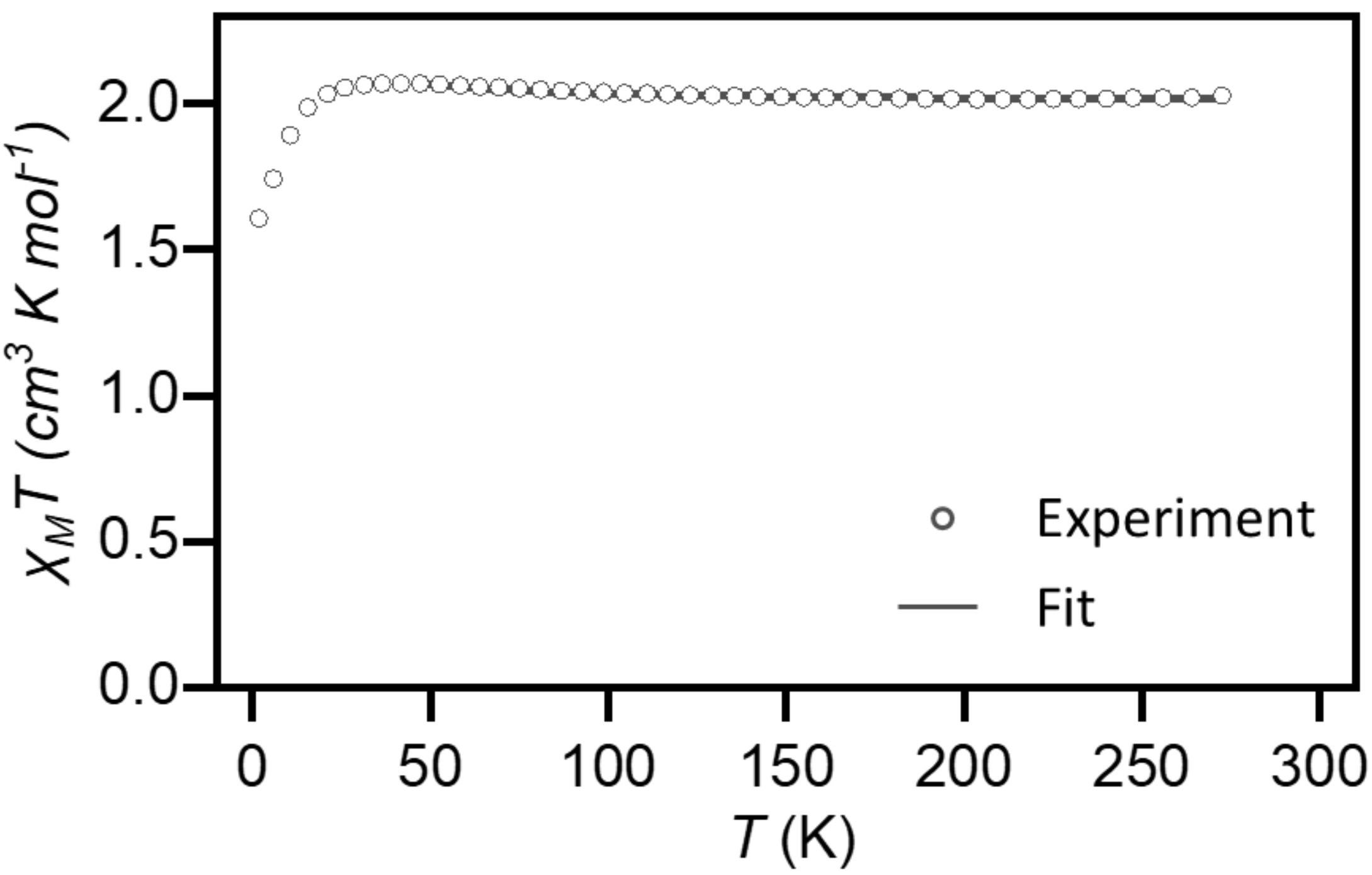

Figure S22. Molar paramagnetic susceptibility-temperature product χ_M of **2** as a function of temperature T recorded by solid-state DC SQUID magnetometry (Measurement setup: T = 1→273 K, H = 500 Oe; μ_{eff} = 4.2(2) μB).

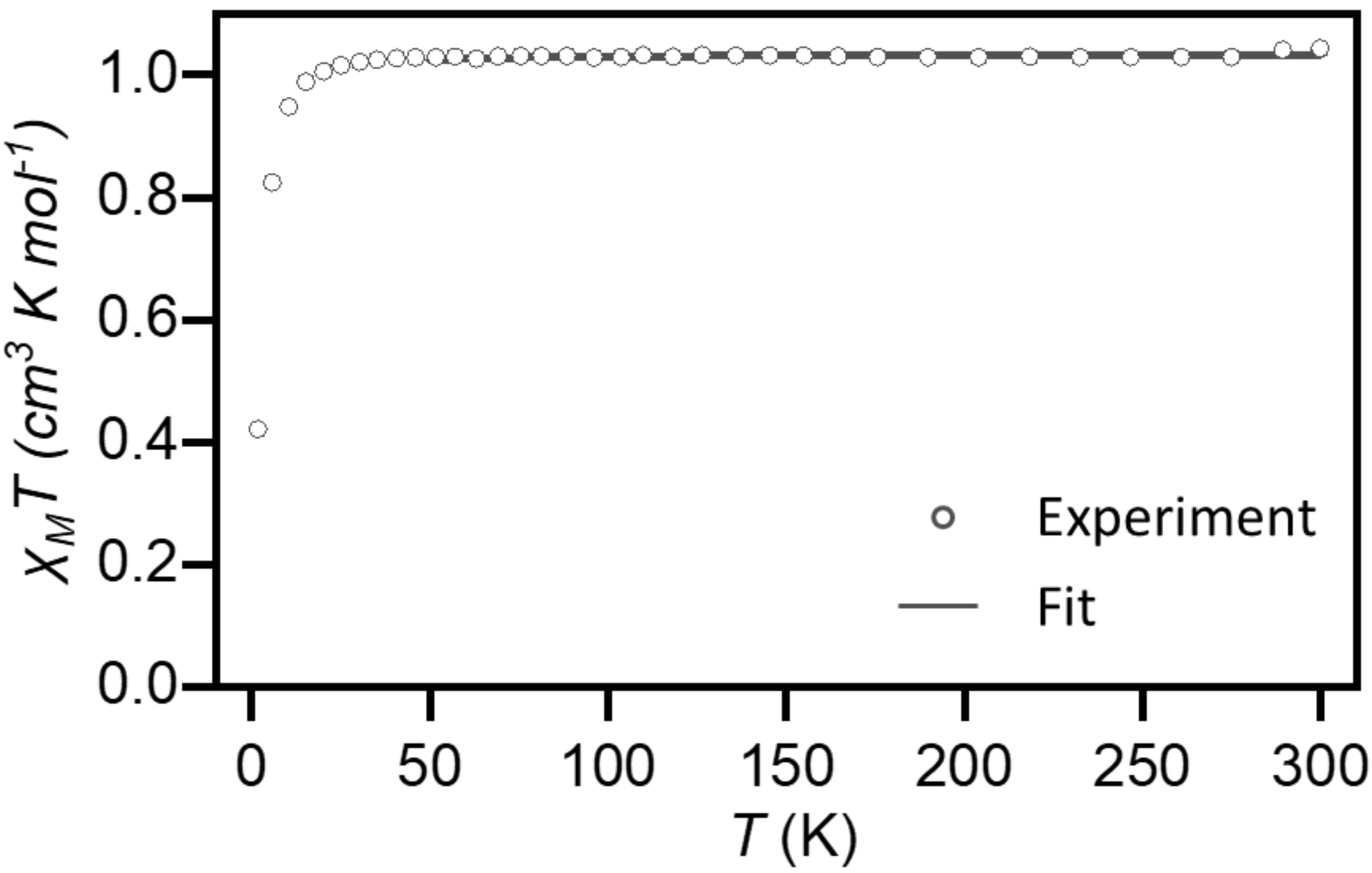

Figure S23. Molar paramagnetic susceptibility-temperature product χ_M of **3** as a function of temperature T recorded by solid-state DC SQUID magnetometry (Measurement setup: T = 1→300 K, H = 2000 Oe; μ_{eff} = 3.0(2) μB).

7. Supplementary Crystallographic Data

Crystallographic data for **2** was measured with Oxford Diffraction Supernova dual-source diffractometer at 150 K using Cu Kα (1.54184 Å). Crystallographic data for **3** was measured with a Rigaku Synergy instrument at 100 K with monochromated Mo−Kα (λ = 0.71073 Å) radiation. The measured data was processed with the CrysAlisPro[7] software package. The structures were solved with the ShelXT[8] structure solution program and refined with ShelXL 2018[8] to convergence against F2. Final refinement was within OLEX.[9] All non-hydrogen atoms were refined using anisotropic thermal parameters and the hydride H atom positions were refined freely. Selected crystallographic and refinement data is given in Table S1. Single crystal diffraction data for are reported in crystallographic information files (CIF) accompanying this document and deposited with the CCDC as deposition numbers **2588736-2588737**. Full details on data collection, reduction and refinement can be found in the individual CIFs. Additional information on modelling disorder of the complexes is outlined below.

7.1. $[(dcype)_2Fe][BAr^F]$ · 1.5 2-MeTHF (**2**)

Highly disordered solvent (2-MeTHF) was found in a pocket located at a crystallographic inversion center. Reflection contributions from the disordered solvent were fixed and added to the calculated structure factors using the SQUEEZE routine of program Platon,[5] which determined there to be 192 electrons in 1000 $Å^3$ that were accounted for in two voids per unit cell. This is consistent with the presence of ½ of 2-MeTHF molecule per asymmetric unit, which accounts for 42 electrons per unit cell.

7.2. $(dcype)_2Fe$ (**3**)

The Fe centre was found disordered over 2 positions with the ratio of 0.81:0.19 and was modelled as such.

Table S1. Selected Crystallographic and Refinement Parameters for compounds **2** and **3**.

**Compound**	**2**	**3**
**CCDC no.**	**2588736**	**2588738**
**Empirical formula**	$C_{99}H_{138}BF_{24}FeO_3P_4$	$C_{52}H_{96}P_4Fe$
**Formula weight**	2022.723	901.082
**Temperature/K**	150.00(10)	100.00(10)
**Crystal system**	orthorhombic	monoclinic
**Space group**	Pnna	$P2_1/n$
**a/Å**	26.5537(4)	10.0653(1)
**b/Å**	20.2914(4)	22.0793(1)
**c/Å**	18.6303(4)	23.0997(1)
**α/°**	90	90
**β/°**	90	97.143(1)
**γ/°**	90	90
**Volume/Å^3**	10038.2(3)	5093.71(6)
**Z**	4	4
**ρ_{calc}g/cm^3**	1.338	1.175
**μ/mm^{-1}**	2.608	3.791
**F(000)**	4268.7	1981.2
**Crystal colour, morphology**	colourless, blocks	orange, blocks
**Crystal sise/mm^3**	0.19 × 0.13 × 0.08	0.38 × 0.31 × 0.23
**Radiation**	Cu Kα (λ = 1.54184)	Cu Kα (λ = 1.54184)
**2Θ range for data collection/°**	7.26 to 148.98	5.56 to 149
**Reflections collected**	34098	119412
**Independent reflections**	10223	10413
**Data/restraints/parameters**	10223/22/578	10413/16/518
**Goodness-of-fit on F^2**	1.019	1.03
**Final R indexes [I>=2σ (I)]**	R_1 = 0.0567, wR_2 = 0.1502	R_1 = 0.0353, wR_2 = 0.0937
**Final R indexes [all data]**	R_1 = 0.0775, wR_2 = 0.1662	R_1 = 0.0379, wR_2 = 0.0952
**Largest diff. peak/hole / e Å^{-3}**	0.99/-0.71	0.50/-0.37

8. References

1. G. Winter, D. W. Thompson, J. R. Loehe, in *Inorganic Syntheses*, **1973**, pp. 99–104.
2. S. Stoll, in *Handbook of Multifrequency Electron Paramagnetic Resonance: Data and Techniques*, S. K. Misra, Ed. (Wiley-VCH, 2014), chap. 3.
3. S. Stoll, *Methods Enzymol* **2015**, *563*, 121–142.
4. S. Stoll, A. Schweiger, *Journal of Magnetic Resonance* **2006**, *178*, 42–55.
5. G. A. Bain, J. F. Berry, *Journal of Chemical Education* **2008**, *85*, 532.
6. E. Kokin, S.-C. Kao, M. J. Kania and M. L. Neidig, Angew. Chem. Int. Ed., n/a, e7300550.
7. 2018, CrysAlisPro Software system, version 1.171.39.46, Rigaku Corporation, Oxford, UK Rigaku Oxford Diffraction Rigaku Oxford Diffraction, **2018**.
8. G. M. Sheldrick, *Acta Crystallographica Section C Structural Chemistry* **2015**, 71, 3–8.
9. O.V. Dolomanov, L. J. Bourhis, R. J. Gildea, J. A. K. Howard, H. Puschmann, *J. Appl. Crystallogr*., **2009** 42, 339-341.
